# Increases in foraging performance of humpback whales in multi-predator feeding aggregations with California sea lions

**DOI:** 10.1098/rstb.2025.0344

**Published:** 2026-10-15

**Authors:** Anna Krylova, Ashley M. Blawas, James A. Fahlbusch, John Calambokidis, Hannah Clayton, Ari S. Friedlaender, Alison K. Stimpert, Jeremy A. Goldbogen, David E. Cade

**Affiliations:** ^1^ College of Science, Northeastern University, Boston, MA, USA; ^2^ Oceans Department, Hopkins Marine Station, Stanford University, Pacific Grove, CA, USA; ^3^ Cascadia Research Collective, Olympia, WA, USA; ^4^ Institute for Marine Sciences, University of California, Santa Cruz Long Marine Laboratory, Santa Cruz, CA, USA; ^5^ San Jose State University, Moss Landing Marine Laboratories, Moss Landing, CA, USA

**Keywords:** foraging efficiency, mixed-species aggregations, prey switching, Monterey Bay, foraging kinematics, fish versus krill feeding

## Abstract

Marine predators frequently form ephemeral multi-predator feeding aggregations around dense prey patches, yet the mechanisms and outcomes of these dynamic events remain poorly resolved. We quantified how California sea lion (*Zalophus californianus*) presence influenced humpback whale (*Megaptera novaeangliae*) foraging using 46 multi-sensor video tags (mean duration: 7.4 ± 7.1 h) deployed on fish-feeding whales in Monterey Bay, California, USA (2014–2023). Across 1808 lunge-feeding events, whales foraging with sea lions exhibited a 23% higher feeding rate and a 16% shorter inter-lunge interval, while the overall percentage of time spent foraging did not change. Across deployments, humpback whale lunge speeds, manoeuvring metrics, and energetic proxies (overall dynamic body acceleration) did not differ with sea lion presence, indicating no detectable increase in per-lunge locomotor cost. When body size was accounted for in a lunge-level linear mixed-effects analysis, sea lion presence was associated with a 35% reduction in approach duration, an 8% increase in approach mean speed and a 25% shorter total lunge duration. Collectively, these results support the interpretation that sea lion-associated bait ball foraging increases humpback whale feeding frequency primarily by compressing search and approach time rather than altering engulfment mechanics or increasing per-lunge energetic expenditure, indicating a potential contribution of sea lions to prey concentration.

This article is part of the theme issue ‘Multi-predator feeding aggregations in pelagic waters’.

## 1. Introduction

The concentration of schooling prey into bait balls can be frenzied, dynamic events that enhance foraging opportunities for multiple predator species [1–7]. The spatially and temporally heterogeneous distribution of resources in the oceans drives predator aggregation around resource hot spots [8–10], in turn increasing predator interactions during foraging events [1]. Temporary heterospecific feeding associations, like those observed among bait ball predators, have been hypothesized to enhance feeding efficiency by facilitating prey detection or capture [3,5]. Mixed species associations (MSAs) have been observed across taxa, including cephalopods, fishes, birds and cetaceans [2,5,11], and where these associations centre on active, co-occurring predation, they may be more specifically characterized as multi-predator feeding aggregations (MPFAs) [12]. In marine ecosystems, a classic example of an MPFA is the interaction between seabirds and dolphins, where dolphins corral bait schools, trapping them at the surface, thus enhancing the diving birds' foraging efficiency [3,6,13]. Such associations may vary widely in outcome, though, ranging from opportunistic co-occurrence to commensal, mutualistic or competitive interactions, depending on ecological context [11,14,15]. Yet, assessing the costs and benefits of MPFAs to individual predators is inherently challenging owing to the difficulty of quantifying the observed dynamics [2,3].

Humpback whales (*Megaptera novaeangliae)* are known for their diverse behavioural repertoire [16–18] and exhibit region-specific foraging adaptability [19–22], prey preferences [23–25] and feeding techniques [26,27]. Documented behaviours include lunge feeding [28], flick feeding [26], lobtail feeding [19], bottom feeding [20], bubble-net feeding [22,27,29] and various forms of cooperative feeding [30–33]. Lunge-feeding, either as the primary strategy or as part of the manoeuvres described above, involves rapidly swimming towards a dense patch of prey, opening the mouth at the edge of the school and engulfing a large quantity of prey at once before subsequent filtering [28].

While these feeding modes are globally diverse, their expression within the California Current System (CCS) is strongly shaped by prey availability and oceanographic variability. At the ecosystem scale, the CCS is highly variable, shaped by cycles of upwelling and oceanographic oscillations that alter prey distributions [34,35]. Recent studies mapping krill density hot spots [36–38] and documenting fluctuating productivity in response to the North Pacific Gyre Oscillation (NPGO) phases and marine heatwaves [34,35,39] underscore the dynamic prey field and ecosystem these whales exploit. Concordantly, humpback whales in the CCS focus on multiple prey types, transitioning primarily between krill (predominantly *Euphausia pacifica* or *Thysanoessa spinifera*) and forage fishes, such as anchovies (*Engraulis mordax*), depending on environmental conditions [23]. This prey switching has been correlated with the NPGO, where positive NPGO phases are associated with increased upwelling and krill abundance, while negative phases favour anchovies [34]. Anchovies are a more energetically rich prey than krill [40], but requires humpback whales to perform more complex manoeuvres and spend longer searching for food between gulps [41]. Therefore, feeding strategies that decrease the time between foraging events should enhance overall foraging effectiveness and would be expected to be adopted rapidly across a population through social interactions [19].

Humpback whales' generalist foraging behaviours are supported by a wide array of morphological and behavioural adaptations. Large pectoral fins are commonly cited as providing a manoeuvrability advantage [21,42,43], while behavioural adaptations like variable lunging speeds [28,41] and prey herding techniques support a humpback whale's ability to exploit diverse prey types [24,28,44]. Together, these physical and behavioural adaptations equip humpback whales to exploit the CCS's shifting prey landscape, positioning them as a key case study for understanding predator resilience under ecosystem variability.

In the CCS, anchovy foraging by humpback whales has increasingly been observed within MPFAs involving California sea lions (*Zalophus californianus*) and dolphins (*Delphinus delphis*) (J. Calambokidis 1986–2025, unpublished data). These encounters, documented through tags and field surveys, often centre on dense anchovy bait balls where multiple predators converge on the shared resource. California sea lions, whose populations have rebounded under the Marine Mammal Protection Act [45], are particularly prominent in these aggregations, forming temporary associations with humpback whales. Still, the underwater mechanisms of these MPFAs remain poorly understood [1,3,14], leaving open critical questions about the relationship of sea lion presence to humpback whale foraging performance.

In this study, we examined the interspecific dynamics between foraging humpback whales and California sea lions in Monterey Bay, California, USA. Specifically, we assessed whether humpback whale feeding rates and locomotor costs changed in the presence of sea lions. Using a decade of tag deployments, we evaluated changes in kinematics and foraging intensity during fish-feeding events. We hypothesized that the presence of sea lions enhances whale prey intake via the elevation of feeding rates. We also predicted that the more chaotic feeding conditions of these MPFAs would demand greater manoeuvring effort, but that any added energetic costs would be offset by increased prey intake.

## 2. Methods

### (a) Archival tag analysis

Short-term (< 1 day) multi-sensor video tags were deployed on 56 fish-feeding humpback whales in Monterey Bay from 2014–2023. Six additional tags from krill-feeding humpback whales were analysed for comparison (2015–2017). Tag deployments were conducted under NMFS permits 26602 and 28850, MBNMS permit MULTI-2025-006 and institutional IACUC protocols using non-invasive suction-cup-attached tags designed by Customized Animal Tracking Solutions (CATS). All tags were deployed from approximately 7 m rigid-hull inflatable boats using a 6 m pole. These tags included tri-axial accelerometers, magnetometers, gyroscopes and pressure sensors. All data were decimated to 10 Hz, then tag orientation on the animal was corrected for and animal orientation was calculated using custom-written MATLAB scripts [46]. Animal speed for all deployments was determined using the amplitude of tag vibrations [47]. In total, 51 of the tags on fish-feeding whales and five of the tags on krill-feeding whales included integrated cameras capturing video at 30 frames s^−1^ [28,48].

### (b) Video and lunge audits

All video recordings (*n* = 125.6 h from 51 fish-feeding deployments; e.g. supplementary material, video S1) were reviewed and scored for the presence of sea lions, sea lion group size, conspecific presence, prey identity and other notable interactions. First- and last-time codes of continuous periods of sightings were recorded, and each continuous period of confirmed sea lion presence or absence was designated as a distinct feeding session for analysis purposes. Screenshots of prey were captured for identification purposes. Lunge feeding events were identified from the kinematic data as periods of acceleration followed by a rapid deceleration, often coupled with significant changes in orientation metrics such as pitch (sensu [28,49]). When available, video observations validated detected lunges.

To integrate ecological data (e.g. surface sea lion activity) with kinematic records, we defined an ‘observation session’ as any continuous period in which the tagged whale foraged under consistent ecological and social conditions: prey type, sea lion presence, and conspecific presence. A session began when these conditions were first established, and a session ended when any one parameter changed (or could not be determined) or when a gap in lunging exceeded 10 minutes. This approach consolidated lunges into analytically meaningful units directly linked to ecological context, while avoiding per-lunge assignments that were sometimes not possible owing to temporary visibility constraints.

To quantify video availability during lunge events, we summarized camera coverage across the 2899 initial lunges identified from kinematic data; 1282 lunges (44.2%) had no video coverage at the moment of engulfment, 1249 lunges (43.1%) had partial but usable coverage (e.g. dark or clipped segments) and 349 lunges (12.0%) had fully clear video during the lunge manoeuvre itself. Video limitations reflected camera angle, tag placement, water clarity, battery constraints and light availability, with footage often ceasing below approximately 50–80 m depth.

Sessions where a condition could not be confidently determined were designated ‘unknown’ and excluded from the audit (rather than being coded as absent). When video was incomplete or unavailable, supplementary information (drone imagery, still photography and contemporaneous field notes) was used to establish conditions for the first consolidated session. Where the sensor data used to determine feeding rates and kinematic parameters extended beyond the duration of video, the ecological and social conditions from the last verified state were provisionally carried forward until a behavioural state change was detected or a greater than 10 minute foraging gap occurred. Analysis of feeding rates and manoeuvrability parameters was thus limited in each deployment to times when sea lion presence could be confidently estimated.

Out of the 56 deployments of fish-foraging humpback whales in Monterey, 46 contained foraging with discernable estimations of sea lion presence, yielding 96 observation sessions integrated in our analysis (supplementary material, table S1). Observation sessions ranged across a wide duration from 1 minute (encompassing a singular lunge) to 14 hours, with a median of 67 minutes and a mean of 147 ± 178 minutes (mean ± s.d.). To calculate feeding rates per unit time, the number of lunges in a feeding bout was divided by the duration of a feeding bout. Feeding bouts, distinct from the observation sessions defined above, for fish-feeding humpback whales were calculated from within this dataset in the same manner as previous studies by examining the distribution of durations between feeding dives (sensu [38]). The threshold found for fish-feeding (5.6 minutes) was similar as in previous studies for krill-feeding (5.5 minutes). Video and lunge audits were then synthesized to generate comprehensive summaries of tag deployments.

Individual whale length measurements were generated by collecting and processing images suitable for photogrammetry of tagged animals (*n* = 17 unique deployments) with a Phantom 4 Pro Advance or similar platform following best common practices [43,49–52] and using publicly available MorphoMetriX 2.0 software available via GitHub (GitHub— MMI-CODEX/MorphoMetriX-V2) [53]. Photo identification information from fluke photography of the tagged whale was included when known, allowing us to note any changes in prey preference of individuals over the course of the study (six deployments = three animals tagged multiple times).

In addition to sea lions, MPFAs in our study area were always characterized by the presence of multiple humpback whales. Because all MPFAs (and 10 out of 15 animals who never fed with sea lions) included conspecific animals, incorporating conspecific presence in statistical models increased Akaike's information criterion (AICs). We were unable to estimate the number of conspecifics present at feeding events from our data, so we could not analyse the effect of group size on feeding rates. However, surface observations indicated that group sizes around anchovy schools ranged from two to approximately 10 humpback whales and approximately 10 to more than 100 sea lions.

### (c) Kinematic analysis

To investigate the energetic expenditure of humpback whales when sea lions were present, individual lunges were analysed in component phases. Lunge feeding is divided into three phases: approach, engulfment, and filtration [54,55]. For our analysis, we included a fourth section distinguishing the approach or searching period from the distinct acceleration that initiates a lunging movement (figures 1 and 2):

− approach: the initiation of the manoeuvring phase before the lunge (run-up phase). Notably characterized by a change and increase in fluke stroke rate, initiation of directed manoeuvring in a pronounced direction and rising baseline speed;
− active lunge: the onset of the lunge attempt, characterized by the primary speed acceleration. The active lunge phase was mapped to the steepest inflexion on the speed curve, with condensed, vigorous strokes, often accompanied by pitch-up and slight roll;
− engulfment: the point from mouth open (maximum speed of the lunge) to mouth closed (the end of the steep part of the speed deceleration; figure 1); and
− filtering: the post-lunge phase where water is expelled through the baleen to trap prey [56]. Characterized by reduced or absent fluke strokes, ending with a noticeable jerk spike or change in acceleration as the whale resumes movement.

**Figure 1.**
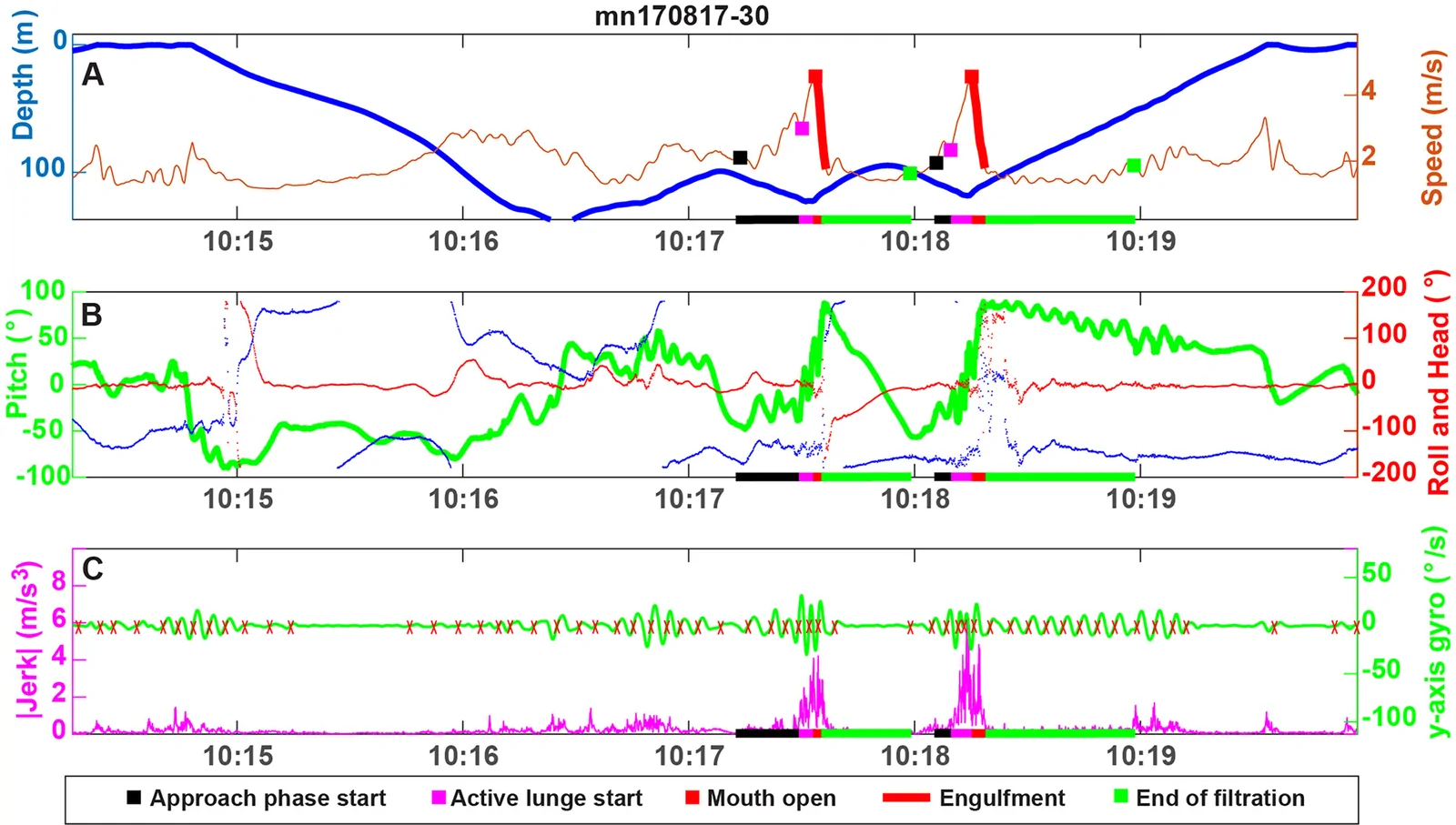
Annotated time series of a double-lunge foraging event by humpback whale mn170817-30, showing the sequential structure and biomechanical phases of two lunges. (A) Depth (blue, left axis) and forward swim speed (brown, right axis). Bottom horizontal bars show colour-coded phase segmentation over time. (B) Three-axis orientation data showing pitch (green), heading (blue) and roll (red) in degrees. Increased angular change occurs during both lunges, particularly in pitch during lunge initiation and roll at terminal mouth opening. (C) Jerk (m s^–^³, pink) and fluke stroke power (green; *y*-axis gyro) illustrating the mechanical effort and acceleration during each lunge. Each stroke is identified with a red x. Peaks in jerk and stroke rate align with engulfment periods. The sequence captures the fine-scale motor patterning and orientation shifts associated with manoeuvring, prey capture and post-lunge gliding.

**Figure 2.**
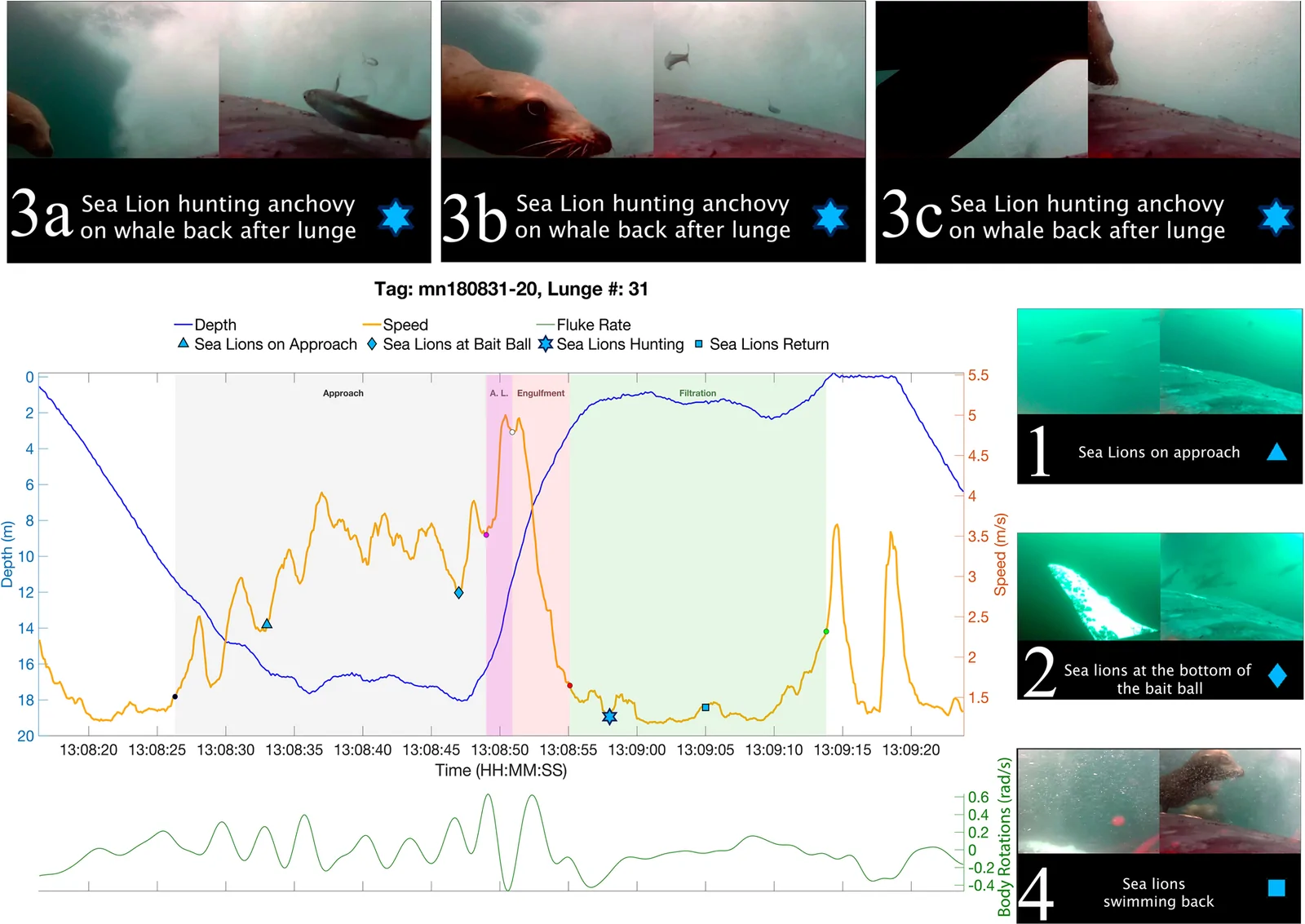
Lunge time series with annotated sea lion interactions during a fish-feeding event (supplementary material, video S1). In panels 3a-c, a sea lion can be seen pursuing escaped prey within the turbulent boundary layer along the tagged humpback whale's back post-lunge (supplementary material, video S1). Coloured markers along the speed plot highlight four key moments of sea lion presence relative to whale behaviour: 1. Sea lions on approach: individuals swimming vigorously during the whale's descent towards the prey school; 2. sea lions at the bait ball: aggregation of sea lions near the prey just prior to the whale's strike; 3. sea lion hunting aftermath: three sequential frames showing a sea lion actively pursuing prey through the whale's turbulent lunge wake; and 4. sea lions return: multiple sea lions swimming back over the whale after lunge completion. Time series layout: lunge foraging phases are shaded: grey, approach; magenta, active lunge; red, engulfment; green, filtration. Phase transition points are indicated by custom markers: blue diamond, approach phase start; magenta diamond, active lunge phase start; white diamond, mouth open; red diamond, mouth close; green diamond, filter phase end.

This kinematic annotation reduced the number of lunges analysed to 1808, as only feeding events with all four component phases clearly identified were analysed. Lunges were excluded from the kinematic analysis if a noisy signal prevented clear determination of phase start or end, or if there was insufficient evidence of a completed lunge (owing to unclear delineation of each phase). Kinematically annotated lunges were compiled to compare the impacts of sea lion presence on humpback foraging through a variety of metrics like speed, manoeuvring and duration (supplementary material, table S2). To examine differences in energetic cost across manoeuvres, we standardized overall dynamic body acceleration (ODBA) for each deployment to the mean of that deployment and used standardized ODBA (henceforth $\hat{\text{ODBA}}$) as a proxy for relative energetic cost [57].

### (d) Statistical analyses

Statistical analyses were conducted using R (Version 4.4.1), RStudio (Version 2024.09.1+394) and MATLAB (Version 2024b) to evaluate the impact of sea lion presence on humpback whale foraging behaviour. Two primary approaches were employed: a tag-deployment-level analysis and a feeding-event-level linear mixed-effects model (LMEM) analysis to account for fixed and random effects like body length and individual deployment. Both sea lion and conspecific presence were recorded as categorical variables (presence–absence).

The first analysis of foraging intensity examined data aggregated by tag deployment. There were 1808 lunges from 46 tags included in this analysis, of which 1109 lunges from 31 of the deployments occurred in the presence of sea lions (61.3%). Deployments were grouped as ‘sea lions present’ if they had more lunges with confirmed sea lion presence than without sea lions present. Averaged values for sea lion presence and absence were compared using two-sample *t*-tests, Welch's *t*-tests (if variance was unequal), and Mann-Whitney *U*-tests (if non-normal). Pooled standard deviations were used when comparing mean metrics across deployments for applicable tests.

The second analysis of lunge characteristics examined the effect of sea lion presence on individual feeding events, while accounting for whale body length and identity, using a LMEM. Because photogrammetric body length measurements were available for only a subset of deployments, this analysis was restricted to 16 tags, contributing 681 lunges, of which 512 (75.2%) occurred when sea lions were present. To account for repeated measures within deployments, tag ID was included as a random intercept. Fixed effects considered included sea lion presence, body length and their interaction. Conspecific presence and temporal covariates (year and month) were also evaluated during model selection.

Candidate model structures were compared across 32 response variables using AIC. The base model structure—sea lion presence, body length, their interaction and a random intercept for tag ID—was retained when additional covariates did not improve model fit. Because conspecific presence strongly overlapped with sea lion presence in this dataset, full statistical separation of these predictors was not possible. Conspecific presence was evaluated as an additional covariate during model selection and not retained. This complementary LMEM approach enabled a finer-scale examination of the influence of sea lion presence on foraging manoeuvres and kinematic mechanisms at the individual, feeding-event level, while simultaneously considering other potential covariate effects that the deployment-level analysis could not resolve. A subset of tags with mixed foraging periods (with and without sea lions) was analysed separately, although the sample size was insufficient for inclusion in the broader analysis.

An *α* of 0.05 was used to assess significance for all statistical tests. All summary values are presented as mean ± s.d. unless stated otherwise. Overall, 43.5% of the deployments occurred in 2018, and several years (e.g. 2015, 2016 and 2021) were under-represented, with only one or two deployments within the year. Tag records were 7.4 ± 7.1 hours in duration, with a mean foraging duration of 3.1 ± 2.8 hours, showing humpbacks spent roughly half of the recorded duration foraging (i.e. the mean foraging ratio was 53 ± 24%). Typical attributes of humpback whale fish lunges were also compared with krill feeding (table 1; supplementary material, table S3).

**Table 1.** Comparison of kinematic and behavioural metrics between krill-feeding (*n* = 7) and fish-feeding (*n* = 45 unless otherwise noted) humpback whale foraging events in Monterey Bay, CA. (Values are mean ± s.d. Statistical comparisons were conducted using Welch's two-sample *t*-tests. Significant differences (*p* < 0.05) are indicated in bold. Relative to krill-feeding events, fish-feeding lunges exhibited longer inter-lunge intervals and greater angular motion during both the approach and active lunge phases (e.g. head, pitch and roll displacement). By contrast, filtration-phase duration and energetic proxies showed no consistent differences between prey types. This table establishes broad prey-based contrasts and provides a baseline for analyses that partition fish-feeding events by sea lion presence (supplementary material, tables S3–S4)).

metric	p-value	krill	fish (all combined)
I: meta information about deployment		(*n* = 7)	(*n* = 45)
**year**	**0.047**	**2016.6 ± 1.3**	**2017.9 ± 2.3**
deployment duration (h)	0.259	10.0 ± 6.2	6.9 ± 6.2
II: lunge phase duration			
**inter lunge interval (s)**	**<0.001**	**119.0 ± 137.5**	**200.5 ± 135.8**
**active lunge duration (s)**	**0.002**	**3.4 ± 2.0**	**5.0 ± 2.7**
**engulf duration (s)**	**0.04**	**3.4 ± 1.0**	**3.0 ± 1.1**
approach duration (s)	0.071	14.4 ± 8.4	16.8 ± 9.1
total lunge duration (s)	0.275	40.2 ± 12.3	44.1 ± 13.8
filter phase duration (s)	0.935	19.1 ± 7.3	19.3 ± 8.3
III: lunge event kinematic parameters			
**approach roll change (°)**	**<0.001**	**69.0 ± 36.2**	**133.9 ± 78.0**
**approach roll change rate (° s^–1^)**	**<0.001**	**5.1 ± 2.1**	**8.5 ± 3.9**
**active lunge roll change (°)**	**<0.001**	**26.8 ± 20.8**	**54.4 ± 37.4**
**active lunge stroke rate (strokes s^–1^)**	**0.002**	**0.2 ± 0.2**	**0.3 ± 0.2**
**approach head change (°)**	**0.005**	**217.0 ± 118.4**	**352.0 ± 180.6**
**active lunge roll change rate (°s^–1^)**	**0.005**	**7.9 ± 4.0**	**11.5 ± 6.7**
**active lunge head change (°)**	**0.006**	**102.9 ± 70.2**	**185.8 ± 116.5**
**approach pitch change (°)**	**0.008**	**139.5 ± 56.1**	**176.2 ± 82.8**
**active lunge pitch change (°)**	**0.009**	**49.9 ± 25.7**	**72.6 ± 40.0**
**active lunge distance (m)**	**0.012**	**10.4 ± 5.8**	**15.8 ± 8.6**
**approach head change rate (°s^–1^)**	**0.038**	**17.0 ± 9.4**	**23.4 ± 11.2**
approach distance (m)	0.052	32.5 ± 17.1	41.2 ± 21.4
filter phase stroke rate (strokes s^–1^)	0.078	0.1 ± 0.1	0.2 ± 0.1
approach mean speed (m s^–1^)	0.092	2.3 ± 0.4	2.5 ± 0.4
active lunge $\hat{\text{ODBA}}$	0.238	7.1 ± 1.4	4.9 ± 1.4 (*n* = 25)
maximum speed (m s^–1^)	0.275	3.4 ± 0.3	3.7 ± 0.6
mouth open speed (m s^–1^)	0.276	3.3 ± 0.4	3.6 ± 0.6
approach $\hat{\text{ODBA}}$	0.283	4.3 ± 1.3	3.3 ± 1.0 (*n* = 25)
approach stroke rate (strokes s^–1^)	0.305	0.3 ± 0.1	0.3 ± 0.1
active lunge head change rate (°s^–1^)	0.313	32.8 ± 18.3	39.9 ± 20.8
active lunge mean speed (m s^–1^)	0.446	3.0 ± 0.4	3.1 ± 0.5
active lunge pitch change rate (°s^–1^)	0.550	15.4 ± 3.7	15.0 ± 5.0
engulf distance (m)	0.652	8.7 ± 2.2	9.0 ± 3.2
approach pitch change rate (°s^–1^)	0.743	10.9 ± 3.2	11.1 ± 3.5
filter phase $\hat{\text{ODBA}}$	0.896	1.0 ± 0.7	1.0 ± 0.6 (*n* = 25)

## 3. Results

### (a) Tag deployment level analysis results

Humpback whales lunged 23% more frequently per foraging hour in the presence of sea lions compared with foraging instances without sea lions (20.9 ± 5.0 versus 17.0 ± 4.7 lunges h^–1^; Mann–Whitney *U* = 122, *p* = 0.001; figure 3). This increase in feeding rate appeared to be partially driven by a 16% decrease in the inter-lunge interval (ILI) (208.5 ± 51.8 versus 175.7 ± 41.1 s) when sea lions were present (two-sample *t*-test, *t*_44_ = 2.39, *p* = 0.021). The overall proportion of time that animals spent foraging (the foraging ratio) was not significantly different between whales foraging with sea lions and whales foraging without sea lions (0.6 ± 0.3 with sea lions, 0.5 ± 0.2 without sea lions, *p* = 0.256).

**Figure 3.**
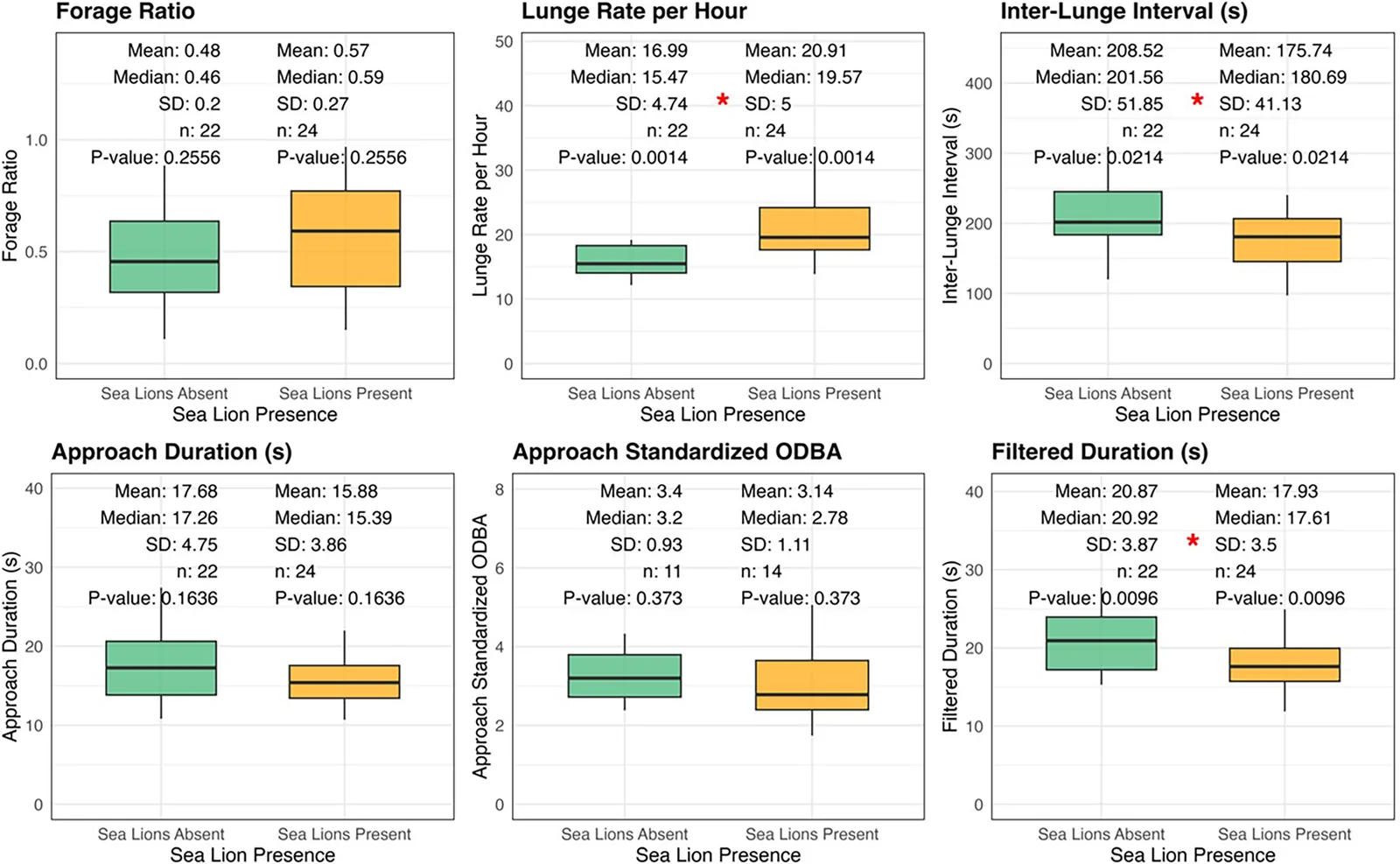
Differences in foraging effort and approach-phase kinematics during humpback whale fish-feeding events with and without sea lion presence. Box plots compare six response variables between events with sea lions absent or present: forage ratio, lunge rate per foraging hour, inter-lunge interval, approach duration, approach standardized ODBA ($\hat{\text{ODBA}}$), and filtration duration. Sea lion presence was associated with higher lunge rates and shorter inter-lunge intervals, indicating increased foraging tempo. Filtration duration was shorter during sea lion-associated events, although this effect was not supported when body size was accounted for (supplementary material, table S5). Asterisks denote significant pairwise differences (*p* < 0.05).

Six deployments had at least three lunges both with sea lions and without sea lions (range of lunges in shorter state: 3–7). This limited statistical analysis and mean differences were not significant, though it is worth noting that in all six cases, lunge rate was higher for foraging with sea lions (mean values 23.5 ± 13.4 lunges h^–1^ versus 16.4 ± 4.7, 95% confidence interval (CI) of difference: –2.4–16.7 lunges h^–1^), and ILI was lower (mean values 177 ± 62 s versus 277 ± 136 s, 95% CI of difference: –216–16 s). Additionally, on two occasions (15 August 2014 and 6 September 2018), one tagged animal fed without sea lions while other tagged animals fed with sea lions. On both days, the animal feeding without sea lions had lower feeding rates and longer ILIs (supplementary material, figure S1).

Whales foraging in the presence of sea lions were observed to have no difference in lunge speeds, duration or distance covered over requisite phases. Sea lion presence was associated with a slightly shorter filtration phase (20.9 ± 3.9 versus 17.9 ± 3.5 s; *t*_44_ = 2.71, *p* = 0.010), paired with a slight increase in filter phase $\hat{\text{ODBA}}$ when foraging with sea lions (1.1 ± 0.2 versus 0.9 ± 0.2, *t*_23_ = –2.54, *p* = 0.018). No differences were observed in manoeuvring metrics in the presence versus absence of sea lions, including orientation changes in pitch or roll, nor stroke rate (supplementary material, table S4), despite the chaotic bait ball environments. Similarly, approach and active lunge $\hat{\text{ODBA}}$ measurements were not different in animals foraging in the presence or absence of sea lions (supplementary material, table S4), indicating no measurable difference in locomotor costs on a per lunge basis associated with sea lion presence. Additionally, there was no difference between $\hat{\text{ODBA}}$ during foraging bouts and periods outside of foraging bouts (recovery times) between whales foraging with sea lions and whales foraging without sea lions (foraging with sea lions was 1.4 ± 0.3 versus 1.3 ± 0.3 without sea lions, *p* = 0.55, 95% CI = –0.2–0.3; time outside of foraging bouts for whales who forage with sea lions was 1.1 ± 0.2 versus 1.0 ± 0.1 without sea lions, *p* = 0.40, 95% CI = –0.1–0.2).

### (b) Linear-mixed effects model results

The lunge-level analysis was limited to the 16 tag deployments with body length measurements (681 lunges total; 512 lunges (75.2%) in the presence of sea lions). Nearly all lunges in this modelling dataset occurred in the presence of conspecific whales, with only a small number (15) of solitary feeding events. To ensure that the observed sea lion effects were not influenced by this small subset, we conducted sensitivity analyses excluding solitary lunges and explicitly evaluated conspecific presence both within non–sea lion feeding events and as an additional fixed effect in candidate models. The pattern of significant sea lion-associated effects remained unchanged, and model support (AIC) generally did not improve with the inclusion of conspecific terms (supplementary material, table S5).

For the majority of response variables (23 out of 32), the top-supported model included sea lion presence and body length as fixed effects, with deployment identity included as a random intercept:

$$\begin{array}{r} \text{response variable}∼\text{sea lion presence}\times \text{body length}+(1|\text{ID}) \end{array}.$$

Temporal random effects (month, year) were evaluated but did not improve model fit and were therefore excluded to avoid unnecessary model complexity.

Approach-phase kinematics differed significantly in the presence of sea lions (supplementary material, table S5). In the base model, approach duration declined by 35% when sea lions were present, decreasing from 22.6 ± 14.6 s without sea lions to 14.6 ± 8.2 s with sea lions (*β* = –60.6, *p* = 0.001). This temporal compression coincided with an 8% increase in approach mean speed, which rose from 2.5 ± 0.5 m s⁻¹ to 2.7 ± 0.6 m s⁻¹ (*β* = 2.6 ± 1.2 s.e., *p* = 0.034). By contrast, approach distance showed a non-significant 27% reduction (54.8 ± 33.2 m versus 39.9 ± 23.9 m; *p* = 0.28). Total pitch change during the approach phase decreased by approximately 32%, from 217 ± 105° to 147 ± 78° with sea lions (*β* = –498.8 ± 160.8 s.e., *p* = 0.003), indicating a more direct and less dynamic approach to prey patches when sea lions were present.

Reductions in approach duration were reflected in shorter overall lunge sequences. Total lunge duration declined by 25%, from 51.9 ± 18.2 s without sea lions to 39.2 ± 13.7 s with sea lions (*β* = –83.9 ± 28.0 s.e., *p* = 0.004). No other manoeuvring variable was significantly impacted by sea lion presence in either level of analysis, suggesting no additional avoidance manoeuvres were conducted when feeding with sea lions, and on average, the same roll and heading manoeuvres were used across both feeding conditions.

Despite these temporal changes, per-lunge energetic and manoeuvring metrics were largely conserved. Active phase lunge speed, distance, duration, stroke rate and orientation changes (heading, pitch and roll) did not differ significantly between feeding conditions. No differences were detected for filter or engulfment phase metrics, including $\hat{\text{ODBA}}$, stroke rates and speed. Together, these results indicate that sea lion presence alters the timing and structure of the approach and total lunge sequence but does not increase per-lunge locomotor or energetic costs, consistent with a shift towards more efficient prey acquisition rather than compensatory effort.

### (c) Opportunistic behaviour observations

Video analyses revealed several behaviours that underscore the flexibility of humpback whale foraging strategies in Monterey Bay. Notably, singular individual prey switching occurred both across years and within single deployments. For example, one individual tagged in 2016 (mn160429-37) fed on krill, but the same whale switched to foraging on fishes in 2018 (mn180424-30). Two individuals (mn140721-3 and mn190725-53) switched from feeding on fishes (as seen on video) in the morning to the more kinematic and behaviourally consistent foraging suggestive of krill feeding [28] later that day. Additionally, benthic sediment interaction, possibly indicative of a third prey type and not known to have been documented in the northeast Pacific, was observed on three deployments (supplementary material, Video S2): mn170410-11 (fish-sediment), mn190725-53 (fish-sediment-krill), and mn210915-58 (sediment only). These observations highlight the potential breadth of humpback whale foraging strategies in Monterey Bay, CA.

Other behaviours of note may warrant further study. Bubble-blowing at depth was documented 19 times across 11 deployments, possibly serving a function in prey corralling or signalling. Sea lions were observed exploiting whale feeding attempts by hunting stunned or escaped fish isolated from the school after lunges (figure 2). Finally, 35 collisions between tagged whales and conspecifics were recorded, with 18 occurring during foraging in the presence of sea lions.

## 4. Discussion

### (a) Foraging advantage of multi-predator feeding aggregations

Humpback whales foraging in association with California sea lions in Monterey Bay exhibited higher feeding rates and shorter inter-lunge intervals than whales foraging without sea lions, without detectable increases in energetic cost per feeding attempt and without significant changes in the proportion of time spent foraging (the foraging ratio, supplementary material, table S4). Specifically, lunge rates increased 23%, from 17.0 to 20.9 lunges per foraging hour, while inter-lunge intervals decreased 16%, from 208.5 to 175.7 s, when sea lions were present. These changes occurred without significant sea lion-associated differences in lunge mechanics or energetic proxies, indicating that individual feeding attempts were not more energetically demanding. Together, these results suggest that humpback whales foraging alongside sea lions achieve higher overall energy gains through increased feeding frequency so long as the prey consumed per gulp (the density of the school) was not diminished in MPFAs (see §4b, prey availability discussion below).

Depending on context and environment, MSAs can arise for a variety of reasons, including foraging advantage, predator avoidance and social or reproductive functions [7,11,14]. The humpback whale–sea lion associations documented here are most consistent with the foraging-advantage hypothesis, placing them within the more specific framework of multi-predator feeding aggregations. Unlike incidental co-occurrences around shared prey, these foraging-advantage MSA events involve repeated, close-proximity exploitation of shared prey, requiring that at least one species actively tolerates, or seeks out, the other's presence for foraging benefit, irrespective of the net outcome for the other party [14,15].

To interpret the ecological significance of these associations, our data are well suited to evaluating outcomes for tagged humpback whales, including feeding rates, kinematics and energetic proxies, but are less able to resolve the origin of each association or to evaluate changes in prey density. The energetic gains from increased feeding rates (table 2) suggest a relationship that may be at least commensal, where humpback whales are better able to find and repeatedly approach dense prey patches. Sea lions, in turn, may profit from dislodged or stunned prey (*sensu*, [59]) after whale lunges (figure 2) and, thus, this relationship may be mutualistic if each species consistently elevates the other's feeding success. Whether whales actively track sea lion activity or both predators simply converge on anchovy schools remains uncertain, and while our data demonstrate that favourable foraging conditions are more common when whales feed in association with sea lions, it is worth further examining the likelihood that sea lions actively increase prey availability for humpback whales, or whether both predators simply converge when prey is more available.

**Table 2.** Hourly energy intake (MJ h^–1^) while actively feeding (during a feeding bout) was estimated for krill-feeding and anchovy-feeding whales, using a representative engulfment volume of 20 m³ (the estimated engulfment volume of an 11.6 m whale, [58]). (Fish density was held constant, although there is an argument to increase this density with hypothesized school packing from sea lion presence. Prey density, capture rates and feeding rate citations are in the table (square brackets). Energy expenditure per lunge was estimated at approximately 1.06 MJ using a published speed-based equation [41]. Results suggest that prey-switching from krill to anchovy—especially when accompanied by sea lion-induced increases in lunge rate—can yield substantial energetic gains (or reduced foraging requirements) for humpback whales.)

prey type	prey density (kg m^–3^)	prey energy content (kJ kg^–1^)	engulfment capacity (m^–3^)	capture rate	feeding rate (lunges h^–1^)	energy gain per lunge (MJ)	hourly energy intake (MJ h^–1^)	percentage difference from typical krill
krill (typical)	0.70 [38]	4220 [40]	20	90% [41]	38 [38]	52	1980	NA
krill (high quality)	0.94 [38]	4220 [40]	20	90% [41]	55 [38]	70	3869	+95% ↑
anchovy (no sea lions)	7.8 [41]	7850 [40]	20	35% [41]	17.0 (this study)	428	7268	+267% ↑
anchovy (with sea lions)	7.8 [41]	7850 [40]	20	35% [41]	20.9 (this study)	428	8936	+351% ↑

### (b) Effect of prey availability

Although we could not measure the density of individual fish schools in this study, prior research has shown that the effect of multiple predators, typically, is to increase the packing density (decrease the space between fish) [e.g. 60–63] of schools, suggesting that each lunge during these MPFAs could additionally involve engulfing more prey per lunge. Sea lions, in particular, have demonstrated herding prey into favourable foraging conditions in other regions [64,65].

In addition to the density of fish in a school (school density), we should also consider whether the number of schools in a foraging region (regional density) or school size could potentially influence the propensity of sea lions and humpback whales to form MPFAs. That is, could an increase in prey quality both explain an increase in interactions between humpback whales and sea lions and our observations of increased feeding frequency? We outline the implications of each of these scenarios in figure 4. Firstly, under all scenarios, optimal foraging theory [66,67] would suggest that an increase in prey quality would lead to a change in the overall time spent foraging as animals optimize their foraging experience. We did not observe a change in the overall proportion of time spent foraging (the foraging ratio, supplementary material, table S4) between whales foraging with sea lions and whales foraging without, suggesting that there was not a substantial difference in general prey availability for the whales foraging with sea lions and the whales foraging without sea lions. In support of this finding, it is worthwhile to consider animals foraging within the same region at similar times (with presumably similar regional prey availability), but with different sea lion occurrences: on the two days when tagged whales foraged simultaneously with differing sea lion presence (supplementary material, figure S1), and in all six scenarios where an animal switched between foraging with sea lions and foraging without, the observations of increased feeding rates and reduced ILIs held.

**Figure 4.**
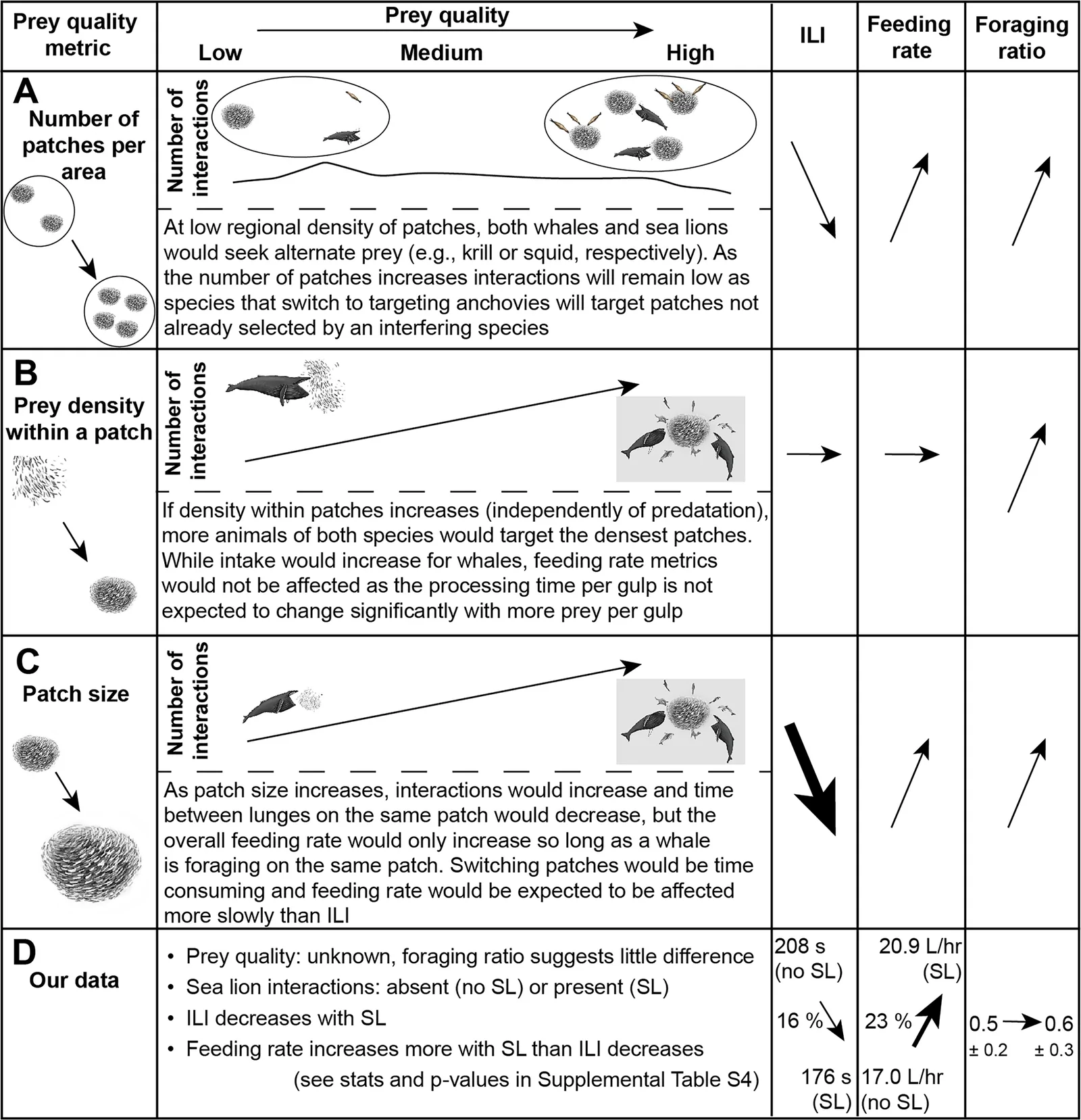
Expected behavioural responses by humpback whales under different kinds of prey quality increase scenarios (A–C) if multi-species aggregations were not beneficial. While prey quality could not be assessed in our current study, the lack of change in foraging ratio suggests similar prey quality in situations where animals foraged with and without sea lions. If prey quality was better when foraging with sea lions despite this observation, the lack of match between our data (D) and the theory suggests that prey quality was not a substantial driver of our observations. Whale and prey illustrations by Alex Boersma, sea lion by Jessica Cohen.

If prey quality changed despite these observations, it is worth considering the three prey quality scenarios outlined above (increases in regional density, school density and school size; figure 4) for our specific ecosystem. Scenario A—increased regional density of fish schools—would be expected to increase the overall number of individuals of both species in an area, but interactions would remain low if the interaction effect was minimal or negative, as species would be motivated to find their own patches rather than share with others. Scenario B—increased school density—indeed is likely to increase the number of foragers of both species; however, as discussed above, increases in school density are probably a result of MPFAs, not a cause. Additionally, increased school density is unlikely to influence ILI or overall feeding rate for whales, as the density of an individual gulp of a whale would lead to greater energetic efficiency and greater consumption per gulp, but the time between gulps (thus ILI and feeding rate) should be unaffected. Scenario C—larger schools—would also lead to more interactions, but the effect on ILI would be substantial (less time between lunges while foraging on a single patch), while the effect on overall feeding rate would be moderate as animals would still have to spend time searching between patches (unless patch number also increased, in which case scenario A applies). We observed the inverse effect (moderate effect on ILI, strong effect on feeding rate).

With these considerations in mind, the most parsimonious explanation of our observations is that prey availability either was not substantially different in our ‘with sea lions’ and ‘without sea lions’ foraging scenarios, or did not have as strong an effect on feeding rate and ILI as sea lion presence, giving credence to the interpretation that sea lions positively influenced humpback whale foraging. One exception to this explanation would be an alternate scenario whereby MPFAs benefit sea lions but not humpback whales, encouraging MPFAs to form even if there was no benefit to humpback whales. Indeed, sea lions have been shown to follow other species, like dolphins [68,69], who can more readily detect or otherwise make schools of prey more readily available. If this alternate scenario were likely, scenario A could be consistent with our observations if increased regional prey density was the driver of the increases in feeding rates. The consistent foraging ratios, the lack of observed antagonistic encounters, and the likely role of sea lions in maintaining school cohesion make this scenario unlikely; however, more research is warranted.

### (c) Monterey Bay as a case study for humpback whale mixed species associations

In the CCS, climate-driven shifts in prey availability have underscored the importance of flexible foraging strategies for species like humpback whales [23]. Within the ecological context of MSAs, Monterey Bay offers a notable case study because, although humpback whales and sea lions overlap elsewhere in the CCS (and globally; supplementary material, figure S2), their joint exploitation of anchovies is pronounced in this region. The CCS itself is highly dynamic, shaped by climate-driven upwelling cycles and ongoing environmental pressures, including prolonged negative phases of the NPGO and intensifying marine heatwaves [34–36]. These processes can destabilize prey distributions, rendering reliance on any single prey item precarious. Anchovies are known to exhibit dramatic boom–bust cycles, as sediment cores spanning centuries reveal an inherent volatility in their populations [39,70–72]. Although both humpback whales and sea lions are broadly generalist feeders [25,26,73,74], anchovies have become an increasingly central resource [75], especially as California's sardine stocks remain historically low [76,77]. A heavy reliance on anchovies, however, can impose significant risks. Poor anchovy availability has been linked to sea lion mass mortalities [74], and reliance on this one species may not be sustainable for humpback whales needing to meet their high-caloric requirements [23,41].

### (d) Mutual advantages of multi-predator feeding aggregations

Against this backdrop of anchovies as a resource with unreliable availability, our findings suggest that the foraging association between humpback whales and sea lions in Monterey Bay may serve to increase the overall availability of anchovies to both humpback whales and sea lions. Sea lions have been documented hunting cooperatively with conspecifics in the Galápagos [64,65] and forming other multi-species foraging aggregations, such as with dolphins or striped marlin (*Kajikia audax*), where they may kleptoparasitize the hunts of ‘producer’ predators [68,69]. Humpback whales, in turn, have been reported associating with killer whales (*Orcinus orca*) in Norway, although that scenario appears largely opportunistic, with whales capitalizing on herring (*Clupea harengus*) herded by orcas rather than deliberately cooperating [78,79]. Humpback whales have also been known to associate with dolphins while foraging, demonstrating an aptitude for joining MPFAs [41,80]. California sea lions primarily feed on schooling fish like anchovy, market squid (*Loligo opalescens*) and sardines (*Sardinops sagax*) [81], and have been observed benefiting from MPFAs with marlin by targeting school fragments that are separated from the main school [69]. Similarly, we observed sea lions foraging on individual, slow-moving anchovies separated from the school by humpback whale lunges (e.g. figure 2). It is also worth noting that the distinct feeding mode of each species—bulk filter-feeding for humpback whales versus single-prey capture for sea lions—may reduce direct competition, thereby facilitating this relatively harmonious co-feeding arrangement. If this association were more competitive, we would expect to see increased manoeuvring expenditure of humpback whales when sea lions are present, as they work to avoid or outmanoeuvre the other species. In the absence of increased manoeuvring, the observed association could therefore reflect a loosely cooperative dynamic, even if not arising from explicit coordination.

In parallel, our findings also align with the ‘rare enemy effect’ [41,82,83], wherein schooling fish adapted to evade solitary, raptorial predators become more vulnerable when confronted by a bulk filter-feeder. This impact may be exacerbated when acting, intentionally or inadvertently, in tandem with a pursuit predator, like sea lions. Although anchovies probably developed schooling as an antipredator defence [60,84,85], the combined foraging efforts of humpback whales and sea lions counteract this strategy by corralling and then disrupting the school, thus nullifying its protective benefit [59]. In other regions, humpback whales achieve a comparable outcome through bubble-net feeding [26,29]; here, abundant sea lions may serve as a functional amplifier of prey density within a school. One hypothesis worth more exploration is whether the joint presence of these two disparate predators creates a dual predation pressure that destabilizes the anti-predation techniques of fish schools. This disruption could render the anchovies more vulnerable than they would be against solitary predators, thereby enhancing foraging success.

### (e) Future directions

Despite these promising insights, several key questions remain unanswered. In particular, it remains unknown exactly how sea lion presence alters the mechanisms of prey aggregation and encounters. Thus, we do not yet understand the drivers of the marked decrease in approach-phase duration (when body length was accounted for) and the inter-lunge interval observed in this study. We propose that these observations could be owing to a corralling effect of the sea lions on the bait ball, allowing the fish school to sustain sufficient minimum prey densities for continued whale foraging. The corralling effect could also work as a signalling aid, reducing the need for searching or manoeuvring for the densest patch of prey, shortening both the approach phase and inter-lunge intervals.

The origin of each bait-ball formation, however, and of humpback-sea lion MSAs more generally, remains uncertain. Bait balls may arise through independent convergence by predators on a pre-existing aggregation, through staggered arrival on the same patch, or through active herding by one or more predators. Each pathway carries different implications for the processes and outcomes, from purposeful coordination to opportunistic co-occurrence to exploitation.

Prey-patch quality also remains unresolved at two scales: the bait ball itself, and the individual engulfment. We do not yet know whether these associations occur only above requisite prey density thresholds, whether sea lions modulate local density, or how basal prey density constrains cases in which humpbacks forage without sea lions. While our data demonstrate higher feeding rates in the presence of sea lions, measurements of engulfed quantities are needed to estimate comparative energetic intake. Relatedly, potential behavioural plasticity in engulfment across prey types or situations, such as krill, schooling fishes and in MSAs, could be evaluated to better understand humpback whale foraging tactics. For sea lions, we lack direct measurements of energy expenditure, realized foraging success and role-specific behaviour within events, so we cannot yet judge whether participation yields net gains or costs to this species. Clarifying initiation, coordination, prey context at both scales and species-specific benefits will help separate the influence of resource quality from interspecific facilitation. A clearer understanding of these factors will refine interpretation of these MPFAs and their kinematic and energetic impacts for these predator-prey dynamics in the CCS.

## 5. Conclusions

In Monterey Bay, humpback whales foraging alongside California sea lions showed higher feeding rates and shorter approach phases without detectable increases in per-lunge energetic cost. Together with targeted video evidence of prey switching (krill, anchovies and a benthic prey) within and across individuals, these results point to a flexible, opportunistic strategy that can capitalize on predator co-occurrence and prey corralling. The mechanism that initiates and sustains these mixed-species events remains uncertain, including origin, the roles of coordination, prey density thresholds and species-specific benefits. Resolving those drivers, through direct measurements of engulfed prey and simultaneous tracking of both predators, will clarify when and how MSAs enhance energy intake for humpback whales, and will improve forecasts of predator responses to a variable CCS.

## Supplementary Material



## Data Availability

Data used for this work include 46 MATLAB-formatted kinematic data files and associated lunge audit files, a behavioural audit spreadsheet noting presence/absences of sea lions and code used to run statistical analyses. These are permanently archived at Stanford University's Digital Repository: https://doi.org/10.25740/cj696yz9785. Supplementary material is available online [86].

## Theme

One contribution of 14 to a theme issue ‘Multi-predator feeding aggregations in pelagic waters’.
