# Supplementary material for: Increases in foraging performance of humpback whales in multi-predator feeding aggregations with California sea lions
Source: Philos Trans R Soc Lond B Biol Sci. 2026 Oct 15;381(1961):20250344. doi: 10.1098/rstb.2025.0344 (PMC13670080; doi:10.1098/rstb.2025.0344)
Supplement: Supplementary file 1 [file rstb.2025.0344.zip › rstb.2025.0344/rstb20250344_si_001.pdf]

Krylova et al. (2026). Increases in foraging performance of humpback whales in multi-predator feeding aggregations

Supplemental Information

Contents:
Supplemental Tables S1-S4
Supplemental Figures S1-S2
Supplemental Videos S1-S2

Supplemental Table S1: Summary of biologging tag deployments with discernible sea lion presence

Deployment ID	Animal ID	Body length (m)	Session count	Video	Photos/notes	Total lunge count	Lunges analyzed	Total feeding time	Lunges with sea lions	Lunges without sea lions	Feeding time with sea lions (high confidence)	Feeding time with no sea lions (high confidence)
mn140716-2b	CRC-10059	NA	1	Y	NA	11	11	0:51:12	0	11	–	0:51:12
mn140716-3	CRC-10250	NA	2	Y	NA	17	11	1:01:12	0	11	–	1:01:12
mn140716-4	CRC-16168	NA	1	Y	NA	4	1	0:16:28	0	1	–	0:16:28
mn140721-3	CRC-11248	NA	4	Y	NA	29	7	1:05:25	1	6	0:14:11	0:51:14
mn140815-2	Unidentified 1	NA	1	Y	NA	72	65	5:17:48	0	65	–	5:17:46
mn140815-3	CRC-15216	NA	1	Y	NA	28	27	1:44:20	27	0	1:44:19	–
mn151012-7	CRC-15273	NA	1	Y	NA	31	27	1:00:25	27	0	1:00:25	–
mn161117-10	CRC-12443	12.71	7	Y	NA	30	14	1:53:54	3	11	0:32:29	1:21:23
mn161117-20	CRC-12093	NA	2	Y	NA	25	21	0:56:25	5	17	0:39:24	0:17:01
mn170410-11	CRC-12297	NA	2	Y	NA	53	47	4:03:25	0	47	–	4:03:24
mn170817-30	CRC-12252	11.69	5	Y	NA	27	21	2:03:32	2	17	0:48:58	1:14:34
mn170817-43	CRC-15481	11.97	3	Y	NA	33	29	2:42:50	7	22	0:34:56	2:07:53
mn170817-50	CRC-10616	13.13	4	Y	NA	95	72	6:17:22	45	3	3:22:06	0:07:15
mn170817-51	CRC-16456	10.6	7	Y	NA	36	29	2:31:08	7	21	2:31:08	–
mn180423-43	CRC-12443	12.71	6	N	Y	207	71	13:14:50	0	0	–	–
mn180424-11	CRC-17791	NA	1	N	Y	69	52	4:14:24	0	52	–	4:14:22
mn180424-30	CRC-16386	NA	5	Y	NA	122	100	4:07:41	0	100	–	4:07:40

mn180607-42	CRC-17789	NA	1	Y	NA	38	21	2:28:50	0	21	–	2:28:49
mn180607-44	CRC-12454	NA	1	Y	NA	14	11	0:54:50	0	11	–	0:54:49
mn180607-45	CRC-16456	10.6	3	Y	NA	226	95	11:52:49	0	95	–	6:45:51
mn180608-47	CRC-12518	NA	3	Y	NA	30	25	2:08:58	0	25	–	2:08:57
mn180807-49	CRC-16615	NA	1	Y	NA	102	98	5:12:02	98	0	5:12:02	–
mn180831-20	CRC-17310	12.38	1	Y	NA	43	29	2:13:09	29	0	2:13:08	–
mn180831-30	CRC-18224	7.82	2	Y	NA	18	12	0:52:58	12	0	0:52:57	–
mn180831-44	CRC-17189	13.1	1	Y	NA	24	21	1:31:51	21	0	1:28:13	–
mn180906-20	CRC-17971	NA	2	Y	NA	16	12	0:38:12	12	0	0:38:12	–
mn180906-30a	CRC-15729	NA	2	Y	NA	11	7	0:25:28	7	0	0:25:28	–
mn180906-30b	CRC-17742	11.65	1	Y	NA	8	7	0:25:25	7	0	0:25:25	–
mn180906-40	CRC-16063	9.99	1	Y	NA	48	44	2:14:57	44	0	2:14:57	–
mn180906-52	CRC-15590	NA	1	Y	NA	6	4	0:22:15	0	4	–	0:22:14
mn181002-45	CRC-15113	12.3	3	Y	NA	190	164	7:55:15	152	0	7:42:40	–
mn181002-47	CRC-15774	NA	1	Y	NA	155	133	4:36:41	133	0	4:33:47	–
mn181002-49	CRC-12434	NA	2	Y	NA	70	47	3:43:06	45	2	3:04:53	0:19:36
mn181002-53	CRC-15286	NA	1	Y	NA	29	25	1:25:34	25	0	1:25:33	–
mn190722-56	Unidentified 2	NA	2	Y	NA	74	65	4:43:40	65	0	4:43:38	–
mn190723-45	Unidentified 3	NA	1	Y	NA	20	19	0:44:05	19	0	0:44:05	–
mn190723-53	Unidentified 4	NA	2	Y	NA	68	60	3:29:02	34	0	1:56:19	–
mn190724-52	CRC-16119	NA	2	Y	NA	12	9	0:38:14	4	5	0:14:06	0:24:08
mn190725-48	CRC-16156	NA	1	Y	NA	24	22	1:18:49	22	0	1:18:48	–
mn190725-53	CRC-12492	NA	5	Y	NA	143	109	6:22:57	16	0	1:38:33	3:06:00
mn190807-52	Unidentified 5	NA	2	Y	NA	111	82	4:28:32	82	0	4:28:32	–
mn210916-49	CRC-16046	NA	1	N	Y	25	23	1:18:14	0	23	–	1:18:13
mn230918-68	CRC-20493	NA	2	N	Y	86	68	5:41:53	0	35	–	2:57:30
mn230925-66	CRC-21169	8.41	3	N	Y	100	86	5:37:54	75	0	5:13:10	–
mn230925-67	CRC-21177	8.82	1	N	Y	46	42	2:39:34	42	0	2:39:34	–
mn230925-68	CRC-21161	7.88	2	N	Y	71	41	5:07:03	41	0	4:39:03	–

Krylova et al. (2026). Increases in foraging performance of humpback whales in multi-predator feeding aggregations

Supplemental Table S2: Metric Names - Descriptions of all kinematic and contextual variables used in the analysis of humpback whale foraging behavior during fish-feeding events, with and without the presence of California sea lions. Metrics span multiple ecological and biomechanical categories, including deployment metadata, prey type classification, behavioral state duration, spatial movement, body acceleration (ODBA), stroke rate, and angular change (head, pitch, roll).

Metric Name	Description
A. Meta information about deployment	
Animal ID	Unique identifier for the individual animal.
Body Length (m)	Measured body length of the animal in meters.
Bout Count	Total number of foraging bouts.
Deployment Duration (hr)	Total time (in hours) that the tag was deployed on the animal.
Forage Duration (hr)	Total duration (in hours) spent actively foraging.
No Sea Lion Bout Duration (min)	Duration (in minutes) of foraging bouts without sea lions present.
Num Bouts with Sea Lions	Number of foraging bouts that included sea lions.
Num Bouts without Sea Lions	Number of foraging bouts that did not include sea lions.
Prey type	Type or classification of prey targeted during foraging.
Sea Lion Bout Duration (min)	Duration (in minutes) of foraging bouts that included sea lions.
Sea Lion Presence	Indicates whether sea lions are present (categorical).
Tag ID	Unique ID for the deployment (mnYYMMDD-TagID)
B. Deployment level comparisons	
Forage Ratio	Proportion of deployment time spent foraging
Lunge Rate per Foraging Hour	Average number of lunges per foraging hour over the deployment
C. Lunge phase duration	
Active Lunge Duration (s)	Mean duration (in seconds) of the active lunge phase.
Approach Duration (s)	Mean duration (in seconds) of the approach phase.
Engulf Duration (s)	Mean duration (in seconds) of the engulfing phase (mouth open and water/prey intake).
Filter Phase Duration (s)	Mean duration (in seconds) of the filtration phase calculated according to body length.
Inter-Lunge Interval (s)	Average time interval (in seconds) between consecutive lunges.
D. Lunge event kinematic parameters	
Active Lunge Distance (m)	Mean distance (in meters) covered during the active lunge phase.
Active Lunge Head Change per second(deg/s)	Mean rate (deg/s) of head-angle change during the active lunge phase.
Active Lunge Head Change(deg)	Mean head-angle change (in degrees) during the active lunge phase.
Active Lunge Mean Speed (m/s)	Mean speed (in m/s) during the "active lunge" phase (A2).
Active Lunge ODBA	Mean ODBA during the active lunge phase.
Active Lunge Pitch Change (deg)	Mean pitch-angle change (in degrees) during the active lunge phase.

Active Lunge Pitch Change per second (deg/s)	Mean rate (deg/s) of pitch-angle change during the active lunge phase.
Active Lunge Roll Change (deg)	Mean roll-angle change (in degrees) during the active lunge phase.
Active Lunge Roll Change per second (deg/s)	Mean rate (deg/s) of roll-angle change during the active lunge phase.
Active Lunge $\widehat{ODBA}$	Mean standardized ODBA during the active lunge phase.
Active Lunge Stroke Rate (strokes/s)	Mean stroke rate (strokes/s) during the active lunge phase.
Approach Distance (m)	Mean distance (in meters) covered during the approach phase.
Approach Head Change (deg)	Mean head-angle change (in degrees) during the approach phase.
Approach Head Change per second (deg/s)	Mean rate (deg/s) of head-angle change during the approach phase.
Approach Mean Speed (m/s)	Mean speed (in m/s) during the "approach" phase (A1).
Approach ODBA	Mean Overall Dynamic Body Acceleration (ODBA) during the approach phase.
Approach Pitch Change (deg)	Mean pitch-angle change (in degrees) during the approach phase.
Approach Pitch Change per second (deg/s)	Mean rate (deg/s) of pitch-angle change during the approach phase.
Approach Roll Change (deg)	Mean roll-angle change (in degrees) during the approach phase.
Approach Roll Change per second (deg/s)	Mean rate (deg/s) of roll-angle change during the approach phase.
Approach $\widehat{ODBA}$	Mean standardized ODBA during the approach phase (a scaled version of ODBA by body length).
Approach Stroke Rate (strokes/s)	Mean stroke rate (strokes/s) during the approach phase.
Depth (m)	Mean depth at which lunging/foraging occurs.
Engulf Distance (m)	Mean distance (in meters) covered during the engulfing phase.
Filter Phase Duration (s)	Mean duration (in seconds) of the filtration or post-lunge filtering phase.
Filter Phase ODBA	Mean ODBA during the filtration phase.
Filter Phase $\widehat{ODBA}$	Mean standardized ODBA during the filtration phase.
Filtration Stroke Rate (strokes/s)	Mean stroke rate during the filtration phase.
Maximum Speed (m/s)	Mean of maximum speeds measured (in m/s) during the lunge.
Mouth Open Speed (m/s)	Mean speed (in m/s) at the moment the animal's mouth is open during a lunge.

Supplemental Table S3: ANOVA comparisons of kinematic metrics across three foraging categories: krill (n = 7), fish without sea lions (n = 27), and fish with sea lions (n = 31). Letters in far right column indicate results of post-hoc Tukey's tests (K = Krill, A = sea lions Absent, P = sea lions Present); groups sharing letters are not significantly different. The presence of sea lions was associated with reduced approach and total lunge duration mirroring that of typical krill lunges in Monterey. Across metrics, krill-feeding lunges exhibited shorter and less variable approach and total lunge durations with reduced angular change relative to fish-feeding lunges, which showed broader, more variable kinematic profiles.

Metric	P value	Krill	Fish (Sea Lions Absent)	Fish (Sea Lions Present)	K	A	P
**Approach Roll Change Rate (°/s)**	0.003	**5.1 ± 2.1 (n=7)**	*7.5 ± 2.8 (n=23)*	9.3 ± 4.6 (n=31)	**b**	*ab*	a
**Approach Head Change Rate (°/s)**	0.008	**17.0 ± 9.4 (n=7)**	*21.9 ± 10.6 (n=23)*	26.1 ± 11.9 (n=31)	**b**	*ab*	a
**Active Lunge Duration (s)**	0.01	**3.4 ± 2.0 (n=7)**	5.1 ± 2.8 (n=23)	4.7 ± 2.6 (n=31)	**b**	a	a
**Filter Phase Stroke Rate (strokes/s)**	0.012	**0.1 ± 0.1 (n=7)**	*0.1 ± 0.1 (n=23)*	0.2 ± 0.1 (n=31)	**b**	*ab*	a
**Active Lunge Roll Change (°)**	0.017	**26.8 ± 20.8 (n=7)**	51.8 ± 29.5 (n=23)	54.6 ± 40.6 (n=31)	**b**	a	a
**Total Lunge Duration (s)**	0.022	**40.2 ± 12.3 (n=7)**	46.5 ± 14.1 (n=23)	**40.8 ± 12.9 (n=31)**	**b**	a	**b**
**Active Lunge Head Change (°)**	0.022	**102.9 ± 70.2 (n=7)**	*182.3 ± 109.7 (n=23)*	195.2 ± 125.6 (n=31)	**b**	*ab*	a
**Inter Lunge Interval (s)**	0.03	**119.0 ± 137.5 (n=7)**	213.7 ± 149.7 (n=23)	203.5 ± 120.6 (n=31)	**b**	a	a
**Approach Roll Change (°)**	0.03	**69.0 ± 36.2 (n=7)**	129.3 ± 64.8 (n=23)	127.0 ± 76.9 (n=31)	**b**	a	a
**Approach Duration (s)**	0.033	**14.4 ± 8.4 (n=7)**	17.8 ± 9.7 (n=23)	**14.8 ± 7.4 (n=31)**	**b**	a	**b**
**Active Lunge Roll Change Rate (°/s)**	0.033	7.9 ± 4.0 (n=7)	10.1 ± 4.6 (n=23)	13.1 ± 7.9 (n=31)	a	a	a
**Active Lunge Distance (m)**	0.034	**10.4 ± 5.8 (n=7)**	16.0 ± 8.8 (n=23)	15.7 ± 8.6 (n=31)	**b**	a	a
**Active Lunge Pitch Change (°)**	0.046	**49.9 ± 25.7 (n=7)**	76.3 ± 44.3 (n=23)	*66.1 ± 35.9 (n=31)*	**b**	a	*ab*
**Approach Head Change (°)**	0.047	217.0 ± 118.4 (n=7)	348.1 ± 168.5 (n=23)	344.2 ± 172.4 (n=31)	ab	a	a
Active Lunge Stroke Rate (strokes/s)	0.078	0.2 ± 0.2 (n=7)	0.3 ± 0.2 (n=23)	0.3 ± 0.2 (n=31)	a	a	a
Filter Phase Duration (s)	0.109	19.1 ± 7.3 (n=7)	20.6 ± 8.3 (n=23)	18.3 ± 8.0 (n=31)	a	a	a
Approach Mean Speed (m/s)	0.142	2.3 ± 0.4 (n=7)	2.4 ± 0.5 (n=23)	2.6 ± 0.4 (n=31)	a	a	a
Engulf Duration (s)	0.143	3.4 ± 1.0 (n=7)	3.0 ± 1.1 (n=23)	3.1 ± 1.0 (n=31)	a	a	a
Active Lunge $\widehat{ODBA}$	0.154	7.1 ± 1.4 (n=7)	5.1 ± 1.3 (n=13)	5.0 ± 1.2 (n=18)	a	a	a
Approach Stroke Rate (strokes/s)	0.158	0.3 ± 0.1 (n=7)	0.3 ± 0.1 (n=23)	0.3 ± 0.1 (n=31)	a	a	a
Approach Pitch Change (°)	0.19	139.5 ± 56.1 (n=7)	180.0 ± 82.1 (n=23)	159.7 ± 72.5 (n=31)	a	a	a
Approach $\widehat{ODBA}$	0.218	4.3 ± 1.3 (n=7)	3.3 ± 1.1 (n=13)	3.3 ± 0.9 (n=18)	a	a	a
Filter phase $\widehat{ODBA}$	0.249	1.0 ± 0.7 (n=7)	0.9 ± 0.5 (n=13)	1.1 ± 0.6 (n=18)	a	a	a
Mouth Open Speed (m/s)	0.277	3.3 ± 0.4 (n=7)	3.5 ± 0.5 (n=23)	3.8 ± 0.6 (n=31)	a	a	a
Maximum Speed (m/s)	0.287	3.4 ± 0.3 (n=7)	3.7 ± 0.5 (n=23)	3.9 ± 0.6 (n=31)	a	a	a
Approach Distance (m)	0.303	32.5 ± 17.1 (n=7)	41.0 ± 21.1 (n=23)	39.0 ± 19.2 (n=31)	a	a	a
Depth (m)	0.348	89.3 ± 27.0 (n=7)	66.0 ± 13.9 (n=23)	67.9 ± 17.9 (n=31)	a	a	a
Active Lunge Mean Speed (m/s)	0.357	3.0 ± 0.4 (n=7)	3.1 ± 0.5 (n=23)	3.3 ± 0.5 (n=31)	a	a	a
Deployment Duration (h)	0.375	10.0 ± 0.0 (n=7)	6.7 ± 0.0 (n=23)	6.5 ± 0.0 (n=31)	a	a	a
Engulf Distance (m)	0.46	8.7 ± 2.2 (n=7)	8.9 ± 3.3 (n=23)	9.6 ± 3.2 (n=31)	a	a	a
Approach Pitch Change Rate (°/s)	0.704	10.9 ± 3.2 (n=7)	10.9 ± 3.8 (n=23)	11.3 ± 3.5 (n=31)	a	a	a
Active Lunge Pitch Change Rate (°/s)	0.718	15.4 ± 3.7 (n=7)	15.0 ± 5.1 (n=23)	14.5 ± 5.0 (n=31)	a	a	a

Krylova et al. (2026). Increases in foraging performance of humpback whales in multi-predator feeding aggregations

Supplemental Table S4 – Two-group comparison of tag-deployment metrics for fish-foraging humpback whales with and without California sea lion presence. Tests (two-sample t-tests or Mann–Whitney U tests, as appropriate) evaluate deployment timing, foraging intensity, phase durations, and lunge kinematics. Whales foraging with sea lions exhibited higher feeding rates and shorter inter-lunge and filtration phases, while overall foraging effort, lunge mechanics, maneuvering, and energetic proxies remained largely unchanged, indicating targeted temporal adjustments rather than increased per-lunge cost.

	Sea lions absent	MSA with sea lions	p value
A. Meta information about deployment			
**Month**	**7.1 ± 2.0 (n=22)**	**8.5 ± 1.1 (n=24)**	**0.006**
**Year**	**2017.1 ± 2.3 (n=22)**	**2018.5 ± 2.1 (n=24)**	**0.009**
Lunge Number in Audit Total	33.4 ± 29.5 (n=22)	52.1 ± 41.8 (n=24)	0.086
Lunge Number in Analysis	53.3 ± 60.1 (n=22)	63.5 ± 49.6 (n=24)	0.286
Forage Duration (hr)	3.2 ± 3.4 (n=22)	3.1 ± 2.2 (n=24)	0.549
Bout Count	9.4 ± 10.0 (n=22)	7.4 ± 5.6 (n=24)	0.699
Deployment Duration (hr)	7.4 ± 7.8 (n=22)	7.5 ± 6.5 (n=24)	0.719
B. Deployment level comparisons			
**Lunge Rate per Hour**	**17.0 ± 4.7 (n=22)**	**20.9 ± 5.0 (n=24)**	**0.001**
Forage Ratio	0.5 ± 0.2 (n=22)	0.6 ± 0.3 (n=24)	0.256
C. Lunge phase duration			
**Filtration Duration (s)**	**20.9 ± 3.9 (n=22)**	**17.9 ± 3.5 (n=24)**	**0.01**
**Inter-Lunge Interval (s)**	**208.5 ± 51.8 (n=22)**	**175.7 ± 41.1 (n=24)**	**0.021**
Approach Duration (s)	17.7 ± 4.8 (n=22)	15.9 ± 3.9 (n=24)	0.164
Active Lunge Duration (s)	5.1 ± 1.3 (n=22)	4.8 ± 1.1 (n=24)	0.306
Engulf Duration (s)	3.0 ± 0.5 (n=22)	3.1 ± 0.5 (n=24)	0.41
Calculated Filtered Duration (s)	22.0 ± 1.4 (n=6)	20.5 ± 3.2 (n=11)	0.513
D. Lunge event kinematic parameters			
**Filtration $\widehat{ODBA}$**	**0.9 ± 0.2 (n=11)**	**1.1 ± 0.2 (n=14)**	**0.018**
Filtration Stroke Rate (strokes/s)	0.1 ± 0.0 (n=22)	0.2 ± 0.0 (n=24)	0.117
Approach Roll Change per second (deg/s)	7.6 ± 2.2 (n=22)	9.1 ± 3.4 (n=24)	0.164
Approach ODBA	0.2 ± 0.0 (n=22)	0.2 ± 0.0 (n=24)	0.2
Active Lunge ODBA	0.3 ± 0.0 (n=22)	0.3 ± 0.1 (n=24)	0.216
Active Lunge Roll Change per second (deg/s)	10.8 ± 4.3 (n=22)	11.7 ± 4.5 (n=24)	0.26
Active Lunge $\widehat{ODBA}$	5.4 ± 1.4 (n=11)	4.7 ± 1.7 (n=14)	0.273
Approach Mean Speed (m/s)	2.4 ± 0.5 (n=22)	2.6 ± 0.4 (n=24)	0.284
Approach Roll Change (deg)	127.8 ± 64.7 (n=22)	133.5 ± 42.0 (n=24)	0.32

Approach Stroke Rate (strokes/s)	0.3 ± 0.0 (n=22)	0.3 ± 0.0 (n=24)	0.33
Approach $\widehat{ODBA}$	3.4 ± 0.9 (n=11)	3.1 ± 1.1 (n=14)	0.373
Active Lunge Pitch Change (deg)	76.3 ± 28.4 (n=22)	70.4 ± 19.4 (n=24)	0.411
Active Lunge Mean Speed (m/s)	3.1 ± 0.7 (n=22)	3.2 ± 0.6 (n=24)	0.454
Engulf Distance (m)	8.8 ± 2.9 (n=22)	9.3 ± 1.7 (n=24)	0.529
Approach Head Change per second (deg/s)	22.8 ± 6.4 (n=22)	24.0 ± 7.2 (n=24)	0.554
Maximum Speed (m/s)	3.6 ± 1.0 (n=22)	3.8 ± 0.7 (n=24)	0.56
Mouth Open Speed (m/s)	3.5 ± 1.0 (n=22)	3.7 ± 0.7 (n=24)	0.573
Active Lunge Roll Change (deg)	53.7 ± 28.9 (n=22)	52.8 ± 16.6 (n=24)	0.624
Approach Pitch Change (deg)	179.9 ± 56.0 (n=22)	173.3 ± 57.9 (n=24)	0.655
Approach Pitch Change per second (deg/s)	11.0 ± 1.9 (n=22)	11.5 ± 2.0 (n=24)	0.687
Active Lunge Stroke Rate (strokes/s)	0.3 ± 0.1 (n=22)	0.3 ± 0.1 (n=24)	0.687
Approach Distance (m)	40.6 ± 11.2 (n=22)	41.4 ± 14.4 (n=24)	0.836
Active Lunge Head Change per second (deg/s)	39.6 ± 13.7 (n=22)	40.1 ± 13.9 (n=24)	0.87
Active Lunge Head Change (deg)	189.8 ± 91.2 (n=22)	180.0 ± 64.9 (n=24)	0.887
Active Lunge Distance (m)	15.8 ± 5.2 (n=22)	15.7 ± 4.3 (n=24)	0.902
Approach Head Change (deg)	353.9 ± 147.2 (n=22)	344.5 ± 115.2 (n=24)	0.922
Active Lunge Pitch Change per second (deg/s)	15.0 ± 3.2 (n=22)	15.4 ± 3.4 (n=24)	0.974

Supplemental Table S5: Summary of lunge-level LMEM outputs for response variables with significant sea lion and/or body-length effects. For each response variable, rows show the observed distribution in the modeling dataset (mean ± SD) for sea lion–absent and sea lion–present lunges, alongside the corresponding LMEM predictions (mean ± SE) for each sea lion condition. Candidate models include the base specification and variants that add temporal random effects (month, year) and/or conspecific terms. Model support is compared using AIC, and fixed-effect p-values are reported for sea lion presence, body length, and their interaction. All sea lion–present lunges in this dataset occurred when conspecific whales were also present.

**Response Variable**	**Model**	**Dataset**	**Mean ± SD (with sea lions)**	**Mean ± SD (without sea lions)**	**Modeled Mean ± SE (with sea lions)**	**Modeled Mean ± SE (without sea lions)**	**AIC**	**p-value (sea lion)**	**p-value (body length)**	**p-value (inter-action)**
Approach Duration (s)	sealion * bodylength + (1|ID)	Filtered (Present Conspecifics)	23.3 ± 14.9	14.6 ± 8.2	22.9 ± 1.5	15.3 ± 0.8	4929	**0.001**	**0.001**	**0.002**
Approach Duration (s)	sealion * bodylength + (1|ID)	Unfiltered	22.6 ± 14.6	14.6 ± 8.2	22.4 ± 1.5	15.3 ± 0.8	5035	**0.001**	**0.001**	**0.002**
Approach Duration (s)	sealion * bodylength + conspecific + conspecific:bodylength + (1|ID)	Unfiltered	22.6 ± 14.6	14.6 ± 8.2	22.8 ± 1.5	15.3 ± 0.8	5038	**0.001**	0.159	**0.002**
Approach Duration (s)	sealion * bodylength + (1|ID) + (1|month) + (1|year)	Unfiltered	22.6 ± 14.6	14.6 ± 8.2	22.4 ± 1.6	14.9 ± 1.0	5038	**0.002**	**0.001**	**0.003**
Approach Duration (s)	sealion * bodylength + conspecific + conspecific:bodylength + (1|ID) + (1|month) + (1|year)	Unfiltered	22.6 ± 14.6	14.6 ± 8.2	22.7 ± 1.6	14.9 ± 1.0	5042	**0.002**	0.157	**0.004**
Approach Mean Speed (m/s)	sealion * bodylength + (1|ID)	Filtered (Present Conspecifics)	2.5 ± 0.5	2.7 ± 0.6	2.6 ± 0.1	2.8 ± 0.1	976	**0.01**	**0.013**	**0.008**
Approach Mean Speed (m/s)	sealion * bodylength + conspecific + conspecific:bodylength + (1|ID)	Unfiltered	2.5 ± 0.5	2.7 ± 0.6	2.6 ± 0.1	2.8 ± 0.1	996	**0.009**	0.791	**0.007**
Approach Mean Speed (m/s)	sealion * bodylength + (1|ID)	Unfiltered	2.5 ± 0.5	2.7 ± 0.6	2.6 ± 0.1	2.7 ± 0.1	999	**0.034**	0.054	**0.031**
Approach Mean Speed (m/s)	sealion * bodylength + conspecific + conspecific:bodylength + (1|ID) + (1|month) + (1|year)	Unfiltered	2.5 ± 0.5	2.7 ± 0.6	2.6 ± 0.1	2.8 ± 0.1	1000	**0.01**	0.777	**0.008**
Approach Mean Speed (m/s)	sealion * bodylength + (1|ID) + (1|month) + (1|year)	Unfiltered	2.5 ± 0.5	2.7 ± 0.6	2.6 ± 0.1	2.7 ± 0.1	1003	**0.034**	**0.054**	**0.031**
Approach Pitch Change (deg)	sealion * bodylength + (1|ID)	Filtered (Present Conspecifics)	219 ± 104	147 ± 78	226 ± 16	156 ± 8	7747	**0.012**	**0.01**	**0.023**
Approach Pitch Change (deg)	sealion * bodylength + (1|ID)	Unfiltered	217 ± 105	147 ± 78	229 ± 15	156 ± 8	7925	**0.003**	**0.002**	**0.007**

Approach Pitch Change (deg)	sealion * bodylength + conspecific + conspecific:bodylength + (1|ID)	Unfiltered	217 ± 105	147 ± 78	225 ± 15	156 ± 8	7928	**0.012**	**0.013**	**0.023**
Approach Pitch Change (deg)	sealion * bodylength + (1|ID) + (1|month) + (1|year)	Unfiltered	217 ± 105	147 ± 78	229 ± 15	156 ± 8	7929	**0.003**	**0.002**	**0.007**
Approach Pitch Change (deg)	sealion * bodylength + conspecific + conspecific:bodylength + (1|ID) + (1|month) + (1|year)	Unfiltered	217 ± 105	147 ± 78	225 ± 15	156 ± 8	7932	**0.012**	**0.013**	**0.023**
Approach Roll Change (deg)	sealion * bodylength + (1|ID)	Filtered (Present Conspecifics)	130 ± 79	134 ± 96	138 ± 18	125 ± 9	7863	0.215	**0.074**	0.214
Approach Roll Change (deg)	sealion * bodylength + conspecific + conspecific:bodylength + (1|ID)	Unfiltered	131 ± 89	134 ± 96	137 ± 17	125 ± 9	8070	0.211	**0.004**	0.208
Approach Roll Change (deg)	sealion * bodylength + (1|ID)	Unfiltered	131 ± 89	134 ± 96	149 ± 17	124 ± 9	8071	0.059	**0.014**	0.061
Approach Roll Change (deg)	sealion * bodylength + conspecific + conspecific:bodylength + (1|ID) + (1|month) + (1|year)	Unfiltered	131 ± 89	134 ± 96	137 ± 18	122 ± 11	8073	0.241	**0.004**	0.247
Approach Roll Change (deg)	sealion * bodylength + (1|ID) + (1|month) + (1|year)	Unfiltered	131 ± 89	134 ± 96	148 ± 17	123 ± 10	8075	0.062	**0.016**	0.066
Inter-Lunge Interval (s)	sealion * bodylength + (1|ID)	Filtered (Present Conspecifics)	239.2 ± 161.7	191.7 ± 138.4	238.6 ± 20.8	200.3 ± 10.9	7865	0.929	0.991	0.815
Inter-Lunge Interval (s)	sealion * bodylength + (1|ID) + (1|month) + (1|year)	Unfiltered	241.3 ± 161.8	191.7 ± 138.4	245.8 ± 21.0	201.8 ± 17.5	8021	0.777	0.847	0.928
Inter-Lunge Interval (s)	sealion * bodylength + (1|ID)	Unfiltered	241.3 ± 161.8	191.7 ± 138.4	238.8 ± 18.9	200.0 ± 10.5	8022	0.571	0.618	0.451
Inter-Lunge Interval (s)	sealion * bodylength + conspecific + conspecific:bodylength + (1|ID) + (1|month) + (1|year)	Unfiltered	241.3 ± 161.8	191.7 ± 138.4	243.6 ± 22.8	199.3 ± 19.0	8023	0.4	0.303	0.494
Inter-Lunge Interval (s)	sealion * bodylength + conspecific + conspecific:bodylength + (1|ID)	Unfiltered	241.3 ± 161.8	191.7 ± 138.4	238.8 ± 20.3	200.1 ± 10.8	8025	0.919	0.382	0.803
Total Lunge Duration (s)	sealion * bodylength + (1|ID)	Filtered (Present Conspecifics)	52.4 ± 17.8	39.2 ± 13.7	50.9 ± 2.7	40.8 ± 1.4	5421	**0.008**	0.017	**0.012**
Total Lunge Duration (s)	sealion * bodylength + (1|ID)	Unfiltered	51.9 ± 18.2	39.2 ± 13.7	51.3 ± 2.5	40.7 ± 1.4	5564	**0.004**	0.011	**0.007**
Total Lunge Duration (s)	sealion * bodylength + conspecific + conspecific:bodylength + (1|ID)	Unfiltered	51.9 ± 18.2	39.2 ± 13.7	50.9 ± 2.7	40.7 ± 1.4	5567	**0.007**	0.125	**0.012**
Total Lunge Duration (s)	sealion * bodylength + (1|ID) + (1|month) + (1|year)	Unfiltered	51.9 ± 18.2	39.2 ± 13.7	51.3 ± 2.5	40.7 ± 1.4	5568	**0.004**	0.011	**0.007**
Total Lunge Duration (s)	sealion * bodylength + conspecific + conspecific:bodylength + (1|ID) + (1|month) + (1|year)	Unfiltered	51.9 ± 18.2	39.2 ± 13.7	50.9 ± 2.7	40.7 ± 1.4	5571	**0.007**	0.125	**0.012**

Krylova et al. (2026). Increases in foraging performance of humpback whales in multi-predator feeding aggregations

Supplemental Figure S1: Within-day timelines of humpback whale lunge feeding in relation to sea lion association.
Panels show same-day deployments from Monterey Bay in which tagged whales foraged on anchovies either in association with California sea lions or in the absence of observed sea lions. Horizontal bars indicate the analyzed foraging-session window for each tag, and points denote individual lunges within that window. The A) 2014-08-15 and B) 2018-09-06 panels provide same-day, same-region contrasts between different individuals with and without sea lions, constraining broad-scale environmental variation while illustrating divergence in foraging timing and lunge pacing. The C) 2017-08-17 example includes both sea lion–associated and non-associated foraging across deployments on the same day, highlighting within-day heterogeneity in lunge structure consistent with variable prey-field organization.

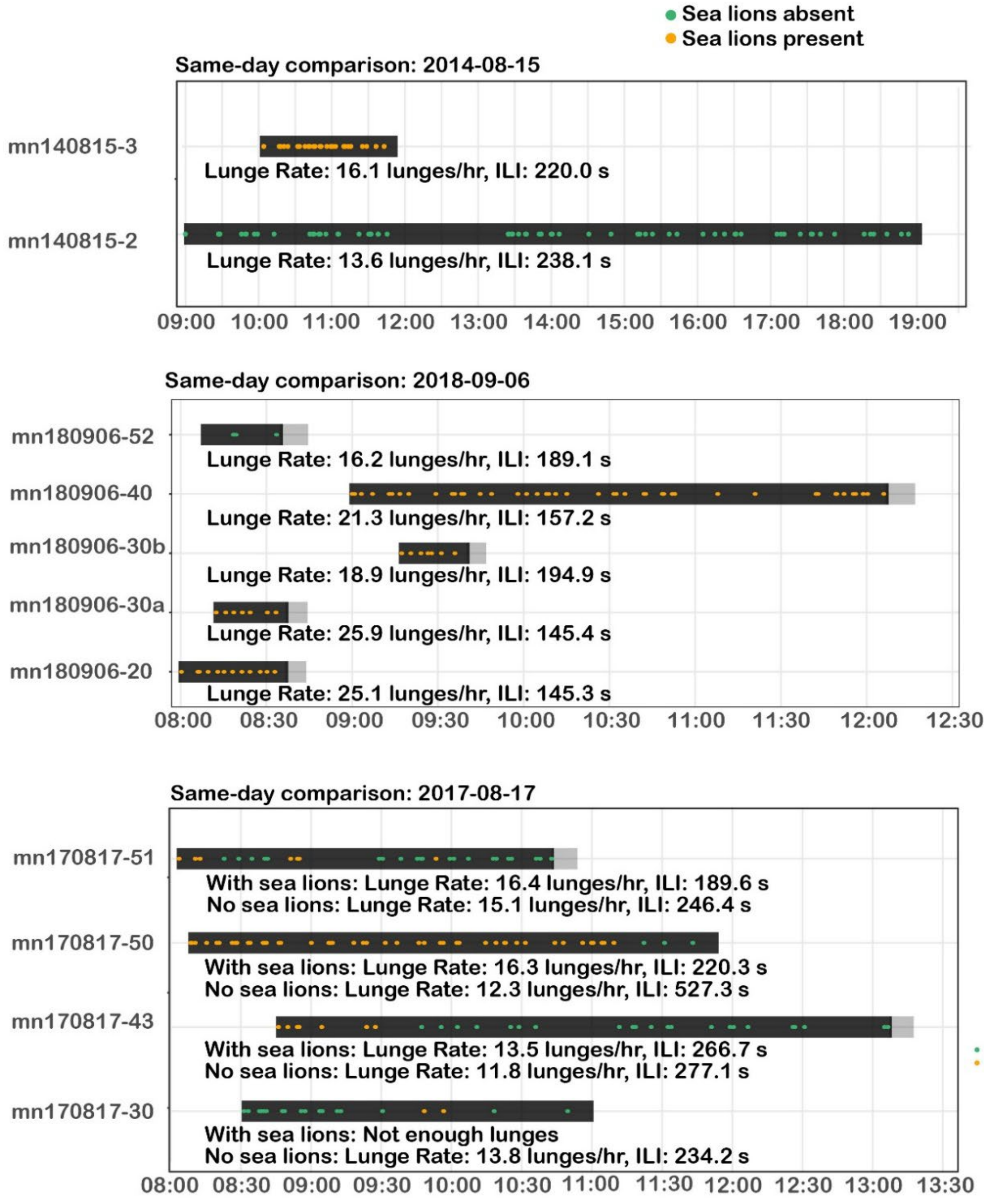

Krylova et al. (2026). Increases in foraging performance of humpback whales in multi-predator feeding aggregations

Supplemental Figure S2 Global distribution maps highlighting spatial overlap between humpback whale habitats (breeding and feeding regions), key forage fish species, and sea lion species.

A) Sea Lion Global Distribution Map

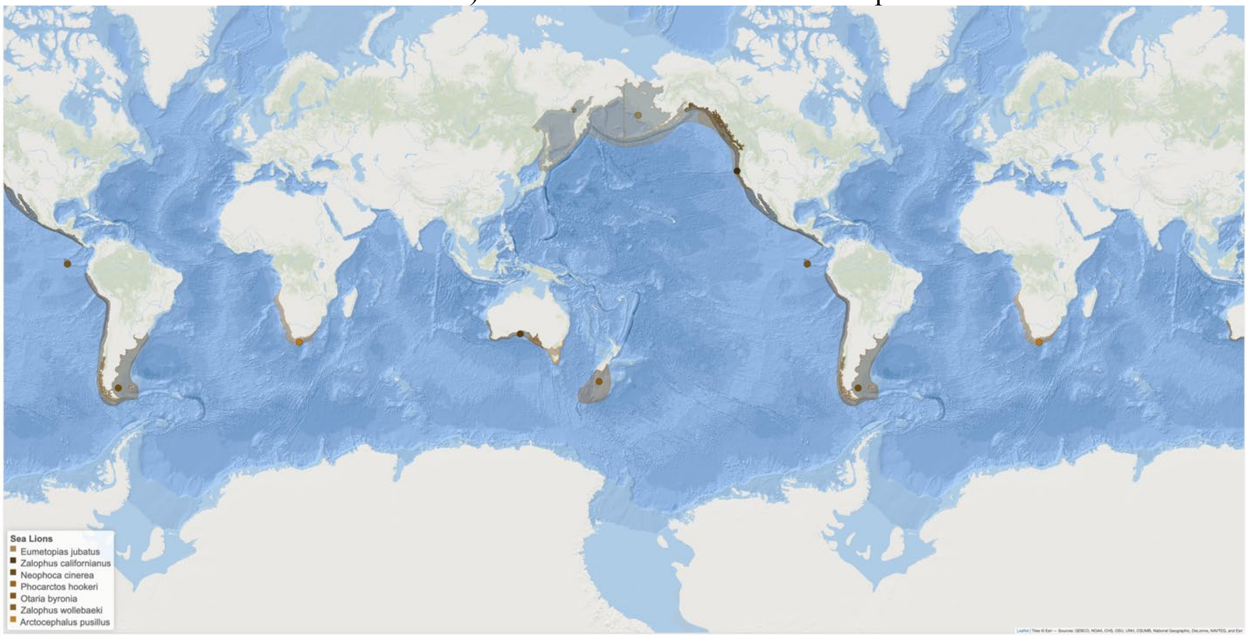

B) Humpback Whale Breeding & Feeding Grounds Map

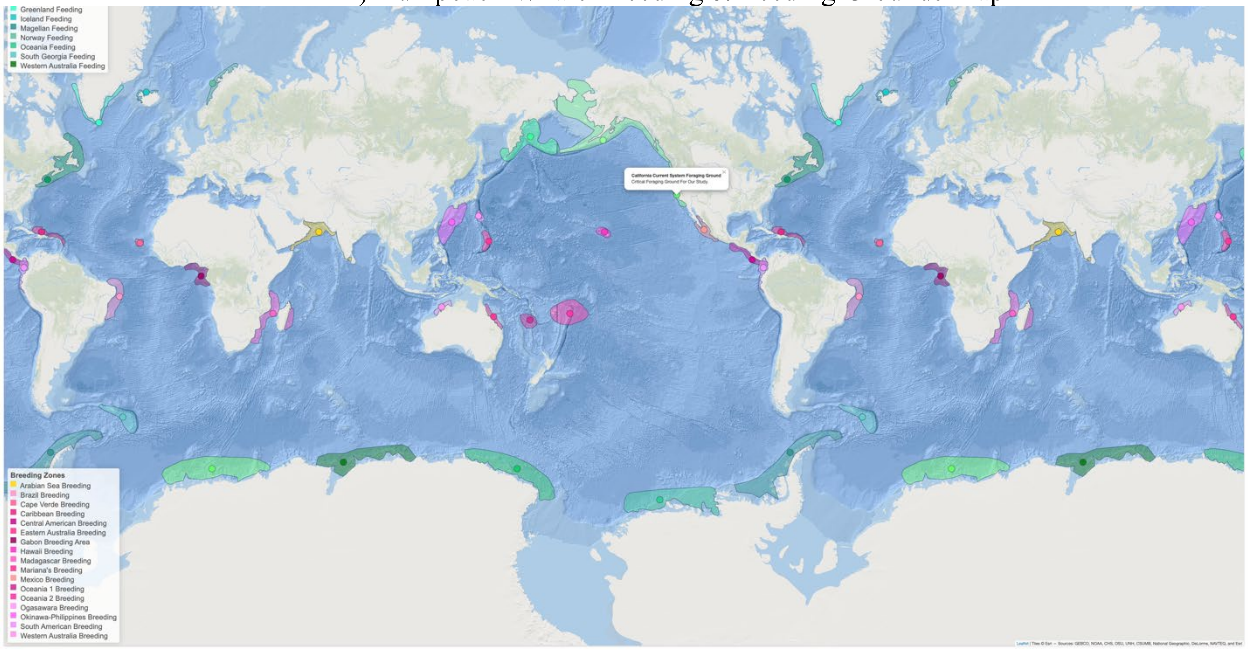

(A) IUCN distributions of seven sea lion species (brown): *Eumetopias jubatus* (Steller sea lion), *Zalophus californianus* (California sea lion), *Neophoca cinerea* (Australian sea lion), *Phocarctos hookeri* (New Zealand sea lion), *Otaria byronia* (South American sea lion), *Zalophus wollebaeki* (Galápagos sea lion), and *Arctocephalus pusillus* (Austral–African fur seal).

(B) Humpback whale breeding areas (pink) and feeding areas (green). *Polygons were hand drawn approximating from various maps to identify key territories and regions associated with different populations.*

C) Anchovy and Sardine Distribution Map

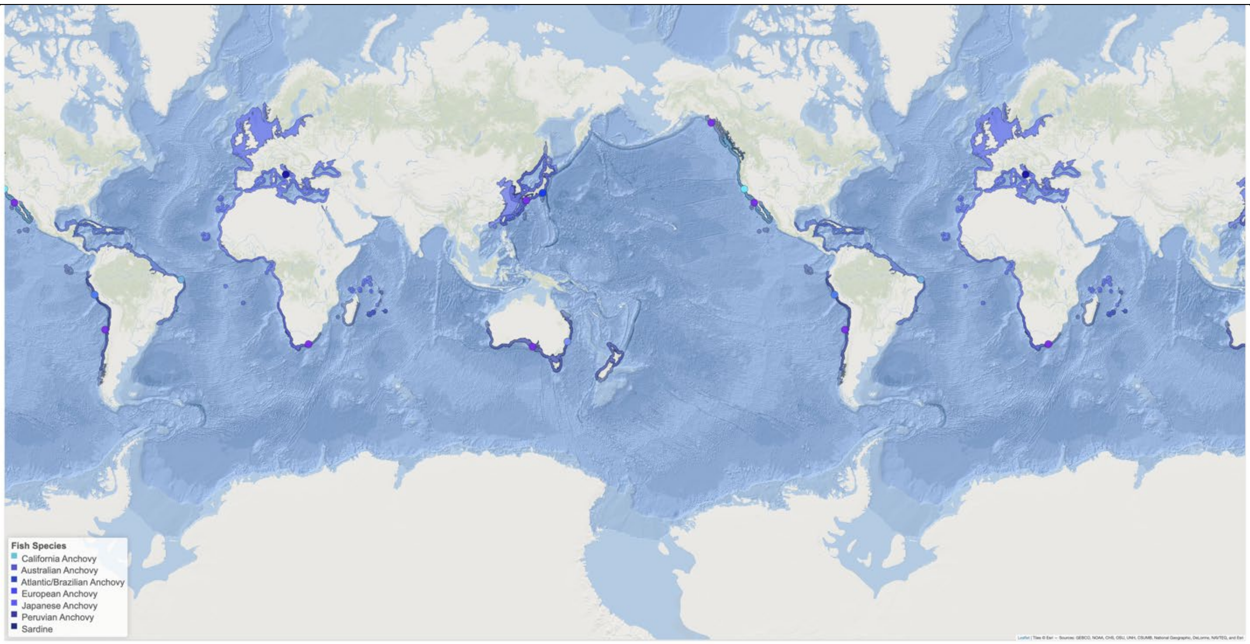

D) Simplified Color Combined Map

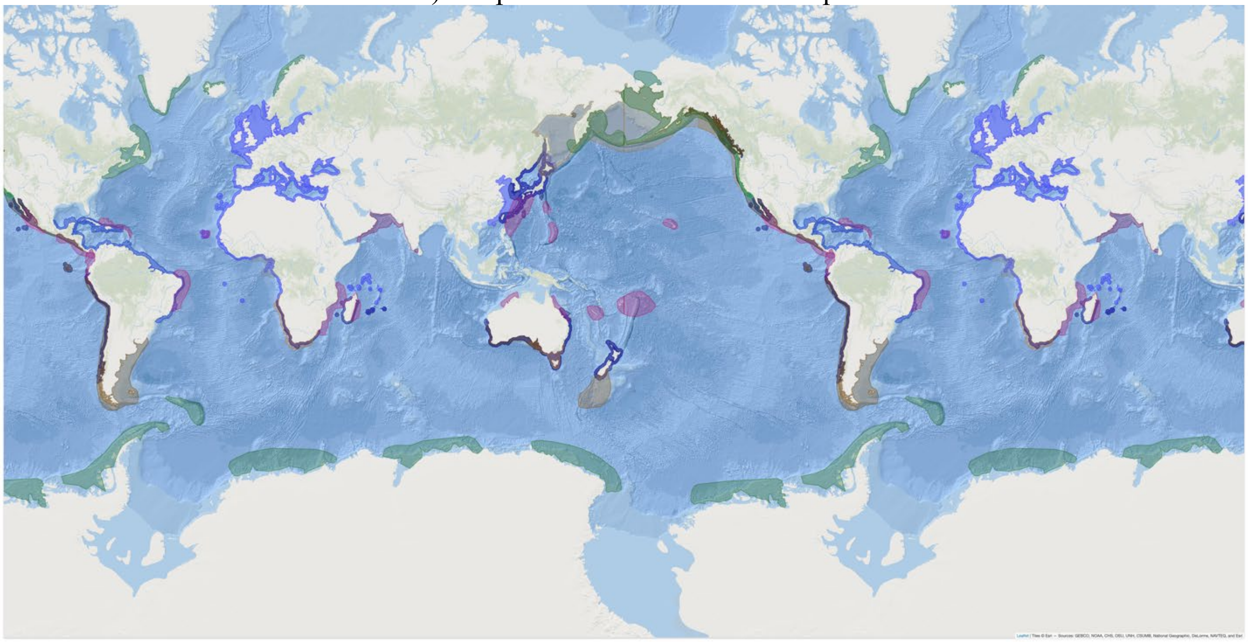

(C) IUCN ranges for seven forage fish species (blue): *Engraulis mordax* (California anchovy), *E. australis* (Australian anchovy), *E. encrasicolus* (European anchovy), *E. ringens* (Peruvian anchovy), *E. japonicus* (Japanese anchovy), *Cetengraulis edentulus* (Atlantic/Brazilian anchovy), and *Sardinops sagax* (sardine).

(D) Final overlay displaying the convergence zones of sea lions, forage fish, and humpback whales, especially in the California Current System (CCS), South American upwelling regions, and the western Pacific. These maps demonstrate the spatial foundations for potential mixed-species foraging associations.

E) Annotated Combined Map

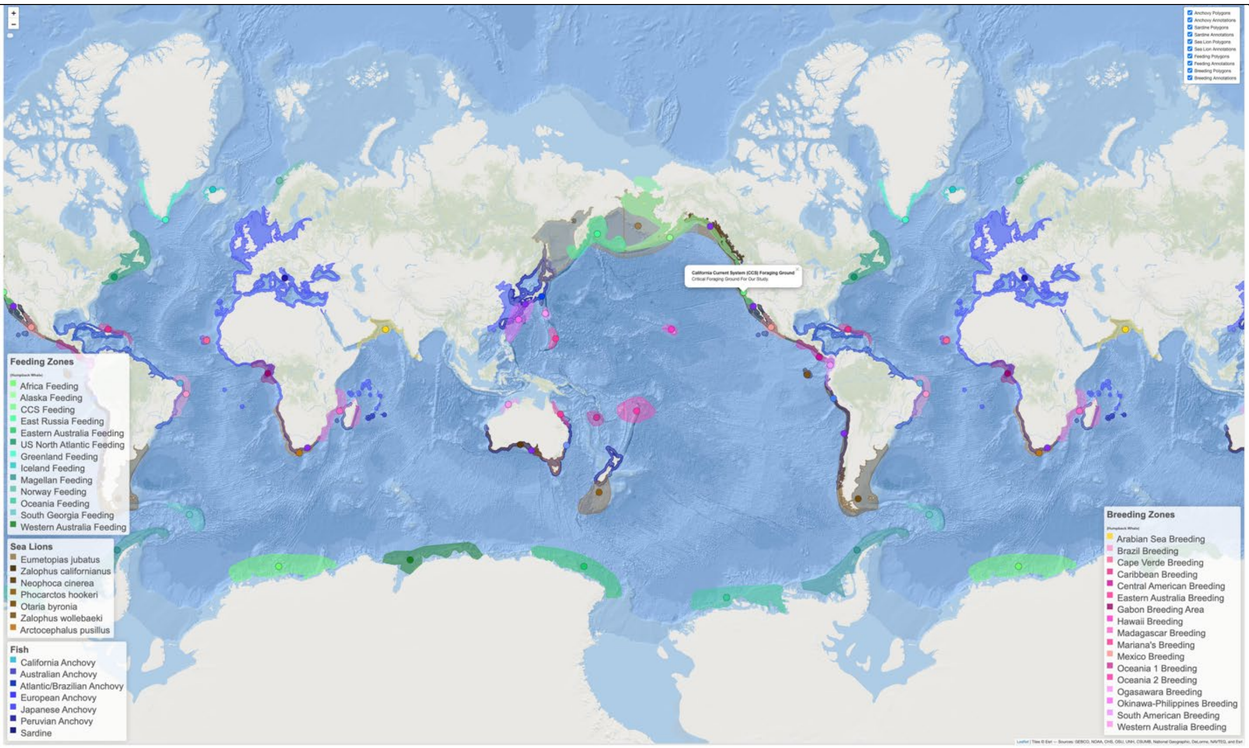

(E) Composite overlay showing all layers together.

Supplemental Video S1
An example video from a CATS tag deployed on the back of a humpback whale showing the approach of the animal and a group of sea lions to a bait ball, a feeding lunge, and then sea lions eating anchovies that were separated from the main bait ball by the lunge.

Supplemental Video S2:
An example video from CATS tags deployed across humpback whales showcasing unknown benthic/sediment interaction with dirt plumes seeming to spill from the jaw area after interaction indicating a possible foraging behavior.
